# Chemical Bonding: The Journey from Miniature Hooks to Density Functional Theory

**DOI:** 10.3390/molecules25112623

**Published:** 2020-06-05

**Authors:** Edwin C. Constable, Catherine E. Housecroft

**Affiliations:** Department of Chemistry, University of Basel, BPR 1096, Mattenstrasse 24a, CH-4058 Basel, Switzerland; catherine.housecroft@unibas.ch

**Keywords:** bonding, valency, affinity, structure, history of science

## Abstract

Our modern understanding of chemistry is predicated upon bonding interactions between atoms and ions resulting in the assembly of all of the forms of matter that we encounter in our daily life. It was not always so. This review article traces the development of our understanding of bonding from prehistory, through the debates in the 19th century C.E. bearing on valence, to modern quantum chemical models and beyond.

## 1. Introduction

Bonding is what separates chemistry from physics. If the understanding of atoms and their component particles belongs primarily to the realm of physics, then chemistry is concerned with the aggregation of atoms into chemical entities held together by *bonds*. If science is a language, and atoms are the letters, bonding is the mechanism by which the letters are combined into words. The International Union of Pure and Applied Chemistry (IUPAC) states that “there is a chemical bond between two atoms or groups of atoms in the case that the forces acting between them are such as to lead to the formation of an aggregate with sufficient stability to make it convenient for the chemist to consider it as an independent ‘molecular species’” [1].

Although the concept of bonding is inherent in the discipline of chemistry and its distinction from its neighbor, physics, our modern understanding of bonding is relatively recent. In the 19th century C.E., as chemists were making enormous advances in the methods for the preparation, purification, and characterization of new molecular species, they were in parallel struggling with developing models for understanding the structure and constitution of these compounds. However, the ideas and concepts did not spring fully-formed into the minds of the 19th century scientists, but can rather be traced as far back as when mankind started to think about matter and its components. This article attempts to give an overview of how we made this journey of discovery and will attempt to demonstrate how the concepts that are today so fundamental that we do not even think about them, proved troublesome and time-consuming to our forebears.

This article is concerned primarily about the roots and origins of our understanding of bonding. Accordingly, it concentrates upon the introduction of new ideas and the controversies and changes that resulted rather than providing a full history of their development after acceptance. The reader is referred to modern texts on bonding [2,3,4,5], in particular the three-volume work by Mike Mingos celebrating the centennial of the chemical bond [6,7,8]. As always, we are indebted to those historians of science who have trodden this path before us in such an able and comprehensive manner [9,10,11,12,13]. A final note concerns the abbreviations of journals; over the years, many journals have changed their titles, some with a confusing and monotonous regularity. For consistency, the CASSI (CAS Source Index) abbreviations are used throughout. In those cases where a journal has changed title, the primary abbreviation is used, even though the original journal title may differ slightly. As an example, the abbreviation *Ann. Phys. (Berlin, Ger.)* is used for the entire period 1819 to date, even though the contemporary title varied amongst *Annalen der Physik und Physikalischen Chemie*, *Annalen der Physik und Chemie*, *Drude’s Annalen*, *Poggendorff’s Annalen der Physik*, *Annalen der Physik (Leipzig, Germany)* or *Annalen der Physik (Weinheim, Germany)*; similarly, the abbreviation *Justus Liebigs Ann. Chem.* is used for *Justus Liebigs Annalen der Chemie*, *Annalen der Chemie und Pharmacie* and *Justus Liebigs Annalen der Chemie und Pharmacie*. After that bibliometric aside, let the journey begin!

## 2. Early Ideas up to the Age of Reason

This section looks at the genesis of atomism and investigates the then contemporary ideas regarding the interactions between atoms that resulted in matter possessing distinct and unique properties. The primary focus of this review is on bonding, but to comprehend how modern views of bonding evolved, we need a broad overview of how our understanding of atoms, which are ultimately the objects that are connected by the bonds, developed. It is convenient and conventional to start this discussion with the philosophies developed by the Early Greek civilization, although this approach is neither chronologically nor historically strictly correct.

### 2.1. The Greeks Had a Word for It

**Figure 1:**
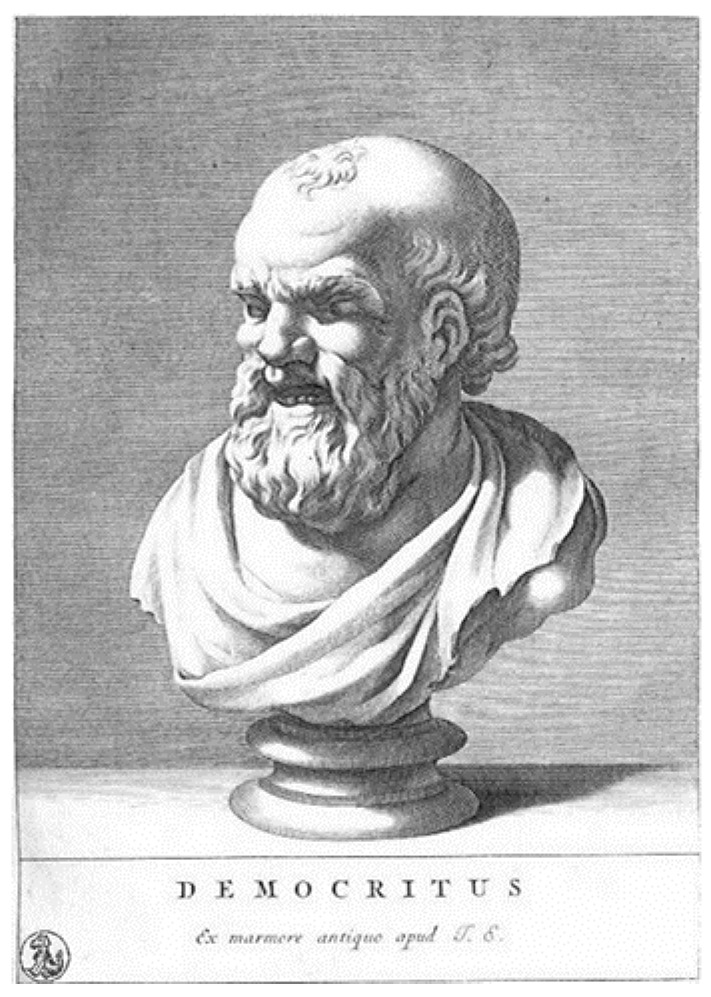
Democritus, together with his mentor Leucippus, are usually credited with the introduction of the atomistic model to Greek philosophy. (Image source, Public Domain, http://www.phil-fak.uni-duesseldorf.de/philo/galerie/antike/demokrit.html).

The word atom (and related terms such as atomic, atomistic, and atomism) is derived from the Greek word: ἄτομος (atomos) meaning indivisible or uncuttable. Most histories of atomism begin with the Greek philosopher Leucippus (Λεύκιππος, Leúkippos) who was supposed to have lived sometime in the fifth Century B.C.E. Very little is known about Leucippus who was credited by Aristotle and Theophrastus as the originator of atomism, although a subsequent champion of the philosophy, Epicurus, maintained that Leucippus was not a historical figure. There is little historical material relating to the philosophy of Leucippus, and his role in the development of atomism is fused irrevocably with the name of his pupil Democritus (Δημόκριτος, ca. 460–ca. 370 B.C.E., Figure 1) [14]. The atomistic philosophy was subsequently refined and extended by Epicurus (Ἐπίκουρος, 341–270 B.C.E.) and Lucretius (Titus Lucretius Carus, ca. 99–ca. 55 B.C.E.). Although only fragments of work that can be ascribed to Leucippus and Democritus survive [15,16,17,18,19], Lucretius’ poem *De rerum natura* (On the Nature of Things) still exists and is a robust didactic defense of atomism [20].

The atomistic philosophy held that the universe was composed of atoms and voids and that matter was composed of these indivisible atoms as building blocks. The void was empty space between the atoms [21]. The fundamental thesis was that if you sub-divided an object a sufficient number of times, you would eventually reach a particle that could not be further divided. These *atoms* were of different types, weights, and shapes, and some possessed hooks or spikes, allowing them to be combined in different ways to generate various types of matter. This is the first physical model that we encounter for chemical bonding. Note that the term atom does not correspond to modern usage; for example, cooking salt (NaCl), stone or wood, was regarded as being composed of atoms of salt, stone, or wood, respectively. Implicit in the atomistic philosophy was the assumption that atoms could be created or destroyed. As Lucretius wrote in *De rerum natura*.

“Two kinds of bodies are to be distinguished: there are primary elements [atoms] of things, and objects compounded of primary elements [atoms]. As for the primary elements, no force has power to extinguish them … The ultimate particles are solid and contain no void … They must of necessity be everlasting.” [22].

The properties of matter were related to the nature of the atoms and their bonding. Hard matter such as metal, stone or diamond is composed of atoms mutually hooked or stuck together in an extended array. Liquids are composed of smooth and spherical atoms, with higher viscosities being related to rougher surfaces or more hooks between the atoms. It must be remembered that early Greek science was not an experimental science as we would recognize, but rather a philosophical construction. There was no question of performing experiments to probe the validity of the atomistic model.

**Figure 2:**
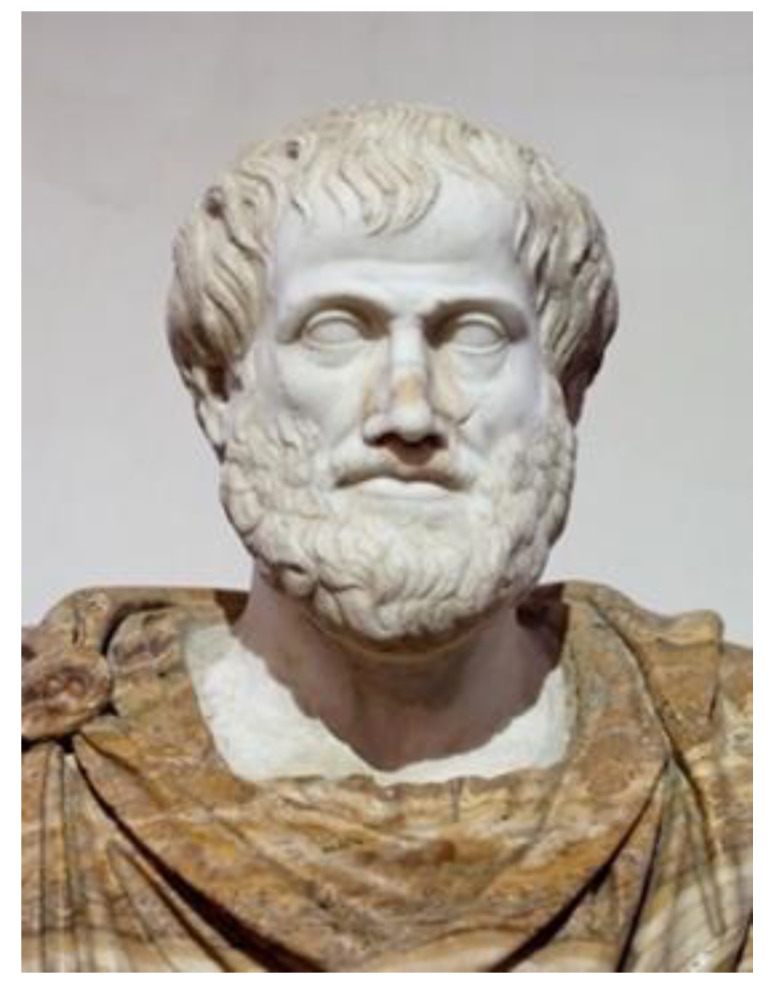
Bust of Aristotle. Marble, Roman copy after a Greek bronze original by Lysippos from 330 BC; the alabaster mantle is a modern addition (Image source, Public Domain, https://commons.wikimedia.org/wiki/File:Aristotle_Altemps_Inv8575.jpg).

The atomistic philosophy was not generally accepted and fell into disrepute in favor of an alternative, originally formulated by Thales (Θαλῆς, ca. 624–ca. 548 B.C.E.). He postulated that all matter was simply a different manifestation of a single basic element, which he subsequently decided was water. The description element does not equate scientifically to our modern understanding of the word, although the concept of a fundamental building block is similar. The philosophy was modified by Anaximenes of Miletus (Ἀναξιμένης ὁ Μιλήσιος; ca. 586–ca. 526 B.C.E.), who suggested that air, not water, was the basic element in an attempt to avoid the need to postulate the existence of a vacuum between material objects, particularly between the Earth and the heavens. In his philosophy, water was a condensed form of air. A third proposal came from Heraclitus of Ephesus (Ἡράκλειτος ὁ Ἐφέσιος, ca. 535–ca. 475 B.C.E.), who suggested that matter was characterized by change and that the basic element was neither water nor air, but fire. It took Empedocles (Ἐμπεδοκλῆς, ca. 494–ca. 434 B.C.E.) to question whether it was necessary to postulate just a single basic element and he developed a philosophy in which matter was composed of the three elements water, air, and fire together with a fourth, earth, that he had added. This is the doctrine of the four elements that we today associate with the philosopher Aristotle (Ἀριστοτέλης, ca. 384–ca. 322 B.C.E., Figure 2). Aristotle also associated the elements with the properties of matter in a philosophy that had a remarkable internal consistency. However, not to be outdone, Aristotle also added a fifth element, ether, which was found in the heavens and is the perfect form of the other four “imperfect” elements. The Aristotelian philosophy is best embodied in the work of Plato (Πλάτων, ca. 428–348 B.C.E.), who equated the shapes of the elements fire, earth, air, and water with those of the “platonic solids”, the tetrahedron, cube, octahedron, and icosahedron. The closest that the Aristotelian model comes to bonding is the rather complex concept of reciprocation [16,23,24].

Aristotle dogmatically rejected the concept that there was any limit to the sub-division of matter, partly because it implied the existence of a vacuum between atoms and in so-doing, essentially condemned the atomistic model to obscurity. The last serious discussions of early atomism that have survived to modern times are attributed to Galen (129–216 C.E.) [25]. Paradoxically, interest in the Aristotelian philosophy in medieval Europe resulted in a rediscovery and revival of the atomistic ideas.

### 2.2. Atomism in Other Early Cultures

Although the emphasis of this review is on bonding rather than atomism, bonding cannot exist without an atomic description and it is interesting to make a short deviation to see how, as so often happens in science, parallel ideas developed in multiple cultures. It seems likely that the ideas of atomism, which are today presented as Greek rationalism were predated by philosophies developed in India and the Middle East.

#### 2.2.1. Indian Atomism

In the eighth century B.C.E., the Vedic sage Aruni (also known as Uddalaka or Uddalaka Aruni), stated that “particles too small to be seen mass together into the substances” [26]. This implies a limit to sub-division of matter to give primal particles and can be seen as the beginning of atomism in the Hindu tradition. Sometime between the sixth and second centuries B.C.E. the Maharishi Kanad (कणाद) proposed an atomistic model of the universe in his book *Vaisheshika Sutra* which was the origin of the Vaisesika philosophy. The philosophy is complex but resembles the later Greek atomism in postulating that matter can only be sub-divided until a smallest entity (anu or paramanu) is reached. The paramanu are indivisible and indestructible. By the seventh century B.C.E. three distinct atomistic schools (the Charvaka, the Jain and the Ajivika) had evolved. Subsequently, the Nyaya and Vaisesika philosophy, developed a model of the universe based upon four basic types of element, reminiscent of the Aristotelian philosophy, but considerably more sophisticated in the relation of the atoms to the properties associated with them. The Nyaya, Vaisesika, and Jaina schools developed bonding models to rationalize the ways in which atoms combined into more complex matter, based upon the formation of dyads and triads [27,28]. This tradition continued well into the modern era with texts as late as the 12th century C.E. discussing atoms made of energy as being point-sized and indestructible [29,30,31].

In parallel to the development of the Hindu atomist philosophy, the Buddhist tradition was developing a world view close to the Greek [32,33]. In the earliest Buddhist tradition, atoms were not regarded as permanent, but by the seventh century C.E., the philosophy had evolved to one of indestructible atoms. The Buddhist philosophy does not seem to have had a well-developed model for bonding between atoms.

#### 2.2.2. Chinese Atomism

It is, perhaps, surprising that China did not develop an atomistic philosophy. Nevertheless, and despite regular cultural, philosophical, and commercial exchange with the Indian sub-continent as well as Buddhist scholars, an atomistic model was not established in the indigenous Chinese natural philosophy. Needham identified fleeting examples of atomistic ideas, but these were never adopted into the mainstream and remained, at best, ephemeral [34]. One can only speculate as to the models and descriptions of bonding that might have been developed.

#### 2.2.3. Islamic Atomism

There is no doubt that the early Islamic philosophers were aware of the atomistic philosophies. At the same time as Buddhist philosophies were being formulated in the 12th century C.E., Islam al-Ghazali (1058–1111 C.E.) was developing the Asharite school predicated upon the postulate that atoms were the only permanent material objects [35]. The Islamic philosophy developed atomism as a consequence of the need for substance to occupy space (*tahayyuz*) and atoms were regarded as indestructible. Convincing arguments have been presented that the Islamic atomistic culture derives from the work of Galen [36]. The Islamic tradition paralleled the ancient Greek, and the atomistic model was rejected by Ibn Rushd (أبو الوليد محمد ابن احمد ابن رشد, Latinized as Averroes, 1126–1198) in favor of a model close to the Aristotelian [37].

### 2.3. The Revival of Atomism in Europe—Entering the Age of Reason

After the decline and eventual fall of the Roman empire, Europe entered a period in which intellectual achievement was less recognized and for which the historical record is lacking. By the beginning of the second millennium C.E., Europe was beginning to look outwards and intellectualize again, in particular through interactions with the Middle East (unfortunately partially typified by the Crusades) and trading of goods along the Silk Road. In addition to the military and commercial exchanges, Europeans were also exposed to new cultures and rediscovered their own forgotten cultural heritage [38].

**Figure 3:**
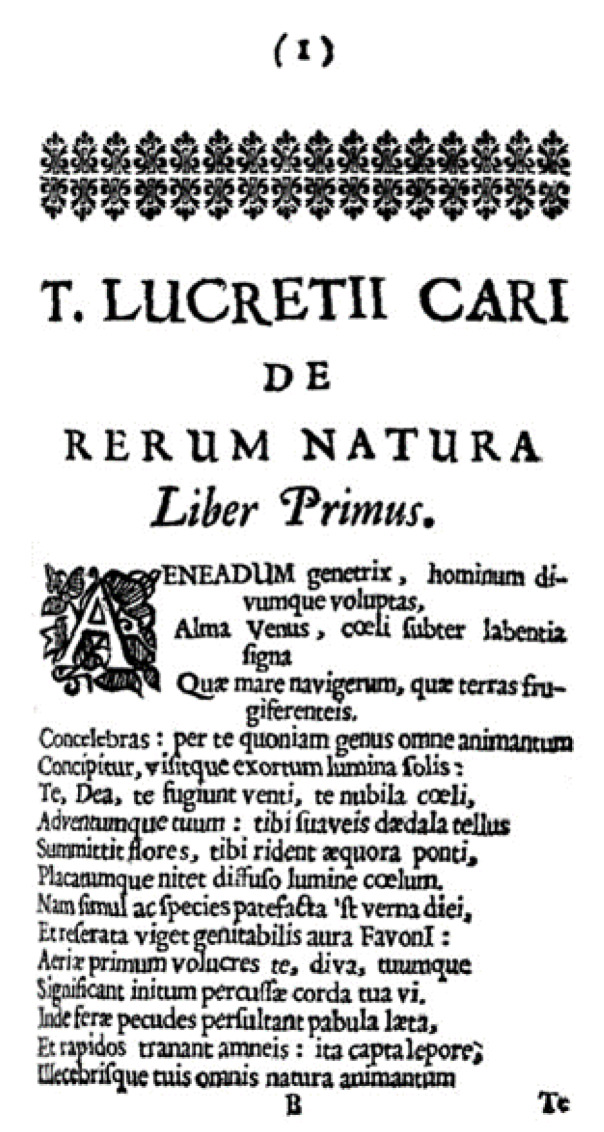
The title page of *De rerum natura* by Lucretius, from the 1675 edition by Tanaquil Faber. (Image source, Public Domain, https://upload.wikimedia.org/wikipedia/commons/6/6e/Lucretius_De_Rerum_Natura_1675_page_1.jpg).

The first wave of this rediscovery can be dated to the period ca. 1250–1350 C.E., when the works of Aristotle were translated from Greek or Arabic into Latin, the scientific language of the day. Paradoxically, the Aristotelian arguments against the atomistic philosophy served to reignite interest in this model in Europe. The implication that matter could be transformed between various forms appealed to both the venal and the scientific nature of man and spawned the mix of mythology and science that we categorize as alchemy. By the 14th century C.E., European scholars had rediscovered the work of Lucretius, in particular *De rerum natura* (Figure 3) and by the 15th century C.E. printed copies had become available to study.

#### 2.3.1. Early Conflicts with Christian Orthodoxy

Lucretius was one of the leading Roman advocates of the philosophy known as Epicureanism (after Epicurus, Ἐπίκουρος, Latinized Epíkouros, 341–270 B.C.E.), which held an atomistic model of the world at its core [39,40,41]. The establishment of Christianity as a Roman state religion by the Emperor Constantine in 313 C.E. resulted in the suppression of Epicureanism (together with its associated atomistic theories), mainly because the philosophy emphasized the neutrality of the gods and their non-interference in the affairs of man. In the early part of the second millennium C.E., atomism remained associated with Epicureanism and in the strongly Christian establishment of the time, atomic models for matter were not intensively studied in Europe.

Galileo Galilei (1564–1642 C.E.) was one of the early scientists who considered atomistic views of matter. Although not directly related to his atomistic views, Galileo had his own differences with the established Christian church regarding his astronomical observations and conclusions [42]. In his 1612 monograph *Discorso al Serenissimo Don Cosimo II¿Intorno alle cose, che Stanno in su l¿acqua, o che in quella si muovono* (A Discourse Presented to the Most Serene Don Cosimo II Great Duke of Tuscany: Concerning The Natation of Bodies Upon, or Submersion In, the Water), Galileo uses an atomistic model to describe the interactions between objects and water [43,44,45]. By 1623, Galileo had further developed his observations into the more coherent atomist form in his publication *Il Saggiatore* (The Assayer), although the philosophy belongs more generally to the school of corpuscularianism, which is atomistic but allows the subdivision of atoms. Certain statements place it specifically in the atomistic tradition rather than that of corpuscularianism, “and perhaps when such attrition stops at or is confined to the smallest quanta, their motion is temporal and their action calorific only; but when their ultimate and highest resolution into truly indivisible atoms is arrived at, light is created” [46,47].

#### 2.3.2. Reconciling Church and Atomism

Pierre Gassendi (1592–1655 C.E.) was a philosopher and scientist who both revived the Epicurean atomistic philosophy and attempted to reconcile it with the Christian church [48,49]. The most complete exposition of the Epicurean atomist view is given in his *Syntagma philosophiae Epicuri* (The Structure of Epicurean Philosophy) published in 1649 [50]. An important feature of the Epicurean atomism was the concept that atoms had “angles and hooks” which allowed them to interact with one another, surely the prototypical description of bonding. One of the earliest Christian refutations of Epicureanism dates to Lucius Caecilius Firmianus Lactantius (c. 250–c. 325 C.E.), who *inter alia* disputed that atoms could have angles and hooks [51]. Gassendi provided a detailed answer to all of Lactantius’ arguments in a robust defense of atomism. Although Gassendi might have been the atomist *par example* at the beginning of the 17th century C.E., he does not appear to have significantly predated John Dalton in giving a modern description of atoms. In his compositionality thesis, Gassendi suggested that combinations of different atoms could assemble into molecules (although this word was not yet in general use for such assemblies), thus defining their macroscopic properties. However, he does not appear to have made the next logical step of associating molecular diversity with the atoms in the combination. Gassendi comes close to developing an atomic or molecular level description that implies affinity (if not bonding) in his discussions of solubility. He explained that *aqua regia* (HNO_3_ and HCl) dissolves gold, and *aqua fortis* (HNO_3_) dissolves silver because the gold atoms fit into the holes between atoms of *aqua regia* and the silver atoms fit into the holes between atoms of *aqua fortis*. One of his greatest contributions was the reconciliation of the atomist view with the orthodox Christian view, perhaps through sophistry, by arguing that everything in the universe, including the atoms, were created by God. Gassendi does not talk about molecules directly, but his description of “seeds of things” (*semina rerum*) equates with Boyle’s *molecula*. Outside the scientific arena, Gassendi is best remembered today for his disputes with his contemporary, the philosopher René Descartes.

It would be disingenuous to attempt to describe the philosophical contributions of Descartes in a few lines and we are here only concerned with his atomistic views. Many of the views presented by Descartes in his *Principles of Philosophy* appear to the modern reader to be very close to those of the Greek atomists [52,53]. There is, however, an important distinction: Descartes believed that matter could, in principle, be infinitely sub-divided. The reflection that this division in principle could (only) be achieved by God provides another point of reconciliation with Christian theology. The bulk of the philosophy is phenomenologically similar to atomism, with the exception that he talks of corpuscles (rather than atoms), which are the basic units that matter is assembled from. This philosophy is also known as corpuscularianism [54,55]. In his views on human perception of matter, Descartes is very close to modern molecular pharmacology, proposing that sensations such as taste are results of the shapes and sizes of the corpuscles or their assemblies. However, for our main topic of bonding, Descartes makes no great contribution as he explicitly rejects bonds between the particles of matter to explain the bulk physical properties. Nevertheless, he does recognize that there must be interactions between corpuscles.

#### 2.3.3. Anti-Aristotleanism and an Early Case for Atomism

As so often happens in science, the time was right for innovation and new ideas. Insomuch as innovation also involves elements of iconoclasm, the views of the influential anti-Aristotelian thinkers are relevant. This school rejected the Aristotelian philosophy in favor of an Epicurean one founded on atomistic thought. This is not the place to examine the conflict that arose regarding these views within the established church. Nevertheless, one particularly influential work published in 1621 is *Philosophiae Naturalis adversus Aristotelem libri XII In quibus abstrusa Veterum Physiologia restauratur et Aristotelis errores solidis rationibus refelluntur a Sebastiano Bassone* by the eponymous Sébastien Basson (1573?–?) [56]. This work systematically demolishes the Aristotelian world view and expounds the atomistic one. However, Basson does not embrace a model in which compounds with different properties are assembled from component atoms, as seen in the work of Sennert (see Section 2.4.1).

#### 2.3.4. An Aside on the Word Molecule

**Figure 4:**
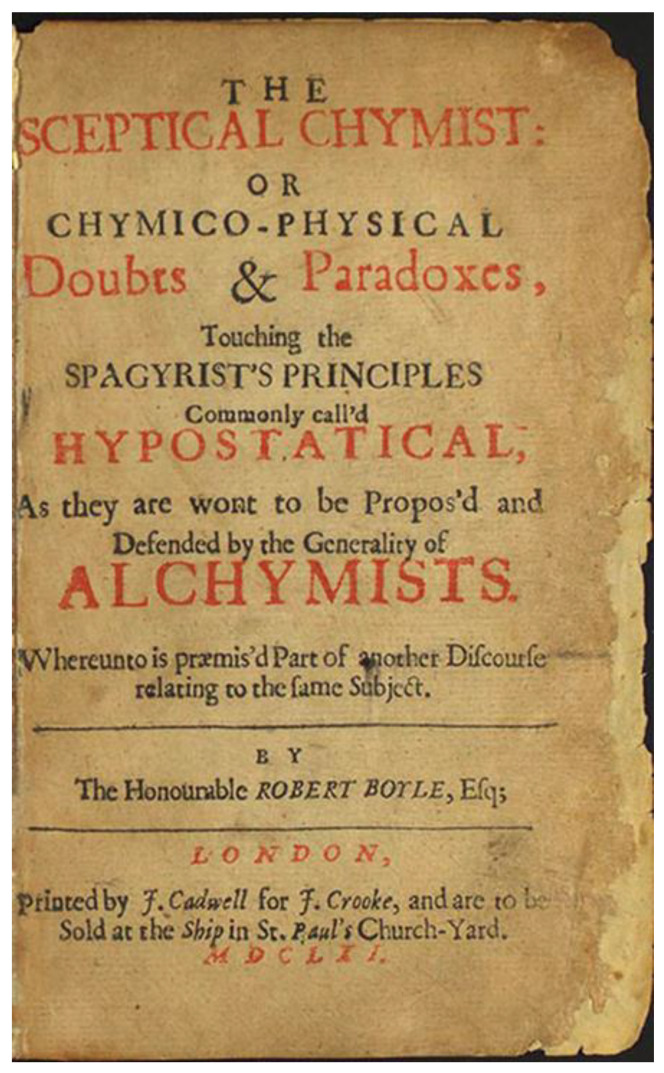
The title page of the 1661 book *The Sceptical Chymist* by Robert Boyle, a pioneering work in the history of chemistry that questioned the Greek notions of elements and Aristotelian atomism (Image source, Public Domain, http://www.gutenberg.org/files/22914/22914-h/22914-h.htm).

In the literature of the 18th century C.E., the words atom and molecule are used with varying degrees of precision. Many authors, including John Dalton, use the term atom to also refer to the smallest unit of a molecular species (for example, atoms of water). At the same time, the word molecule was beginning to be used for discrete species composed of atoms (in the modern sense). Most sources trace the etymology to the French word *molécule*, which in turn is derived from the New Latin *molecula* which is purported to be a diminutive of the Latin *moles*, meaning a mass. One of the earliest uses of *molecula* in a sense that modern readers would understand dates to January 1666 in correspondence between Robert Boyle and D. Coxe, “These Subtilized principles meeting together may bee readily united: which Substances thus united Constitute a little masse, or molecula of mettall, many of which are usually associated before they appeare in a visible or sensible forme” [57]. Interestingly, Boyle does not use the word in his *Sceptical Chymist* of 1661 (Figure 4, *vide infra*).

### 2.4. Early Ideas in the Scientific Age

The transition into the scientific age is a rather arbitrary boundary that we have made on the basis of individuals who the modern mind perceives of as scientists rather than philosophers or alchemists—*mea culpa*. We have also now restricted the discussion to those atomists who explicitly made consideration of chemical bonding and interactions between the atoms. In general, no distinction will be made between atomists and corpuscularianists.

One of the early scientific converts to atomism (or at least to some of its aspects) was Francis Bacon, a philosopher, politician, statesman, and scientist. In his 1612 work *De Principiis atque Originibus* he considers and rejects the alchemical ideas of transformation and argues for atomism or corpuscularianism [58,59,60]. However, in later work, Bacon argued that the bulk properties of matter arose from the size and motion of corpuscles rather than individual atoms and eventually rejected the doctrine of atoms using an Aristotelian argument relating to the absence of a vacuum in nature [61]. Despite his chemical and alchemical credentials, Bacon does not appear to have speculated significantly on the nature of chemical bonding.

#### 2.4.1. Daniel Sennert and a Rational Atomism

One of the early contributions that identify interactions between atoms comes from Daniel Sennert (1572–1637 C.E.) who published his *Hypomnemata Physica* in 1636 [62]. This remarkable document provides an excellent overview of the 17th century C.E. interpretation and incorporation of the Greek ideas into the contemporary philosophy. The debate between atomists and corpuscularianists is moderated by a hierarchical organization of matter. In her excellent assessment of his contributions, Michael [63] summarizes Sennert’s views on atoms and bonding:

(1) The fundamental units of matter are extremely minute bodies called atoms, each of which has a specific substantial form.
(2) The simplest atoms are atoms of the elements. Each is indivisible and immutable.
(3) There are various grades of atoms, and each higher grade of atom is composed of an organization (or structure) of the next lower grade of atoms.
(4) Each body that is not an atom is composed of bonded atoms

Sennert’s elements are the Aristotelian big four—earth, air, fire, and water. Atoms of the “modern” elements, compounds and materials are at the higher organizational levels. This is about as good as it is going to get, in the absence of a modern definition of an element. Sennert described the level of organization corresponding to molecules as *prima mista* and postulated that *prima mista* could be converted back to atoms (although these may have been the Aristotelian atoms).

#### 2.4.2. Robert Boyle—The End of Alchemy or the Beginning of Chemistry?

For chemists, Robert Boyle (1627–1691 C.E.) is immortalized in Boyle’s law:

P_1_V_1_ = P_2_V_2_

which relates the volumes of a gas measured at different pressures at a given temperature. However, he was a very accomplished scientist who made significant contributions to a wide range of chemical and other scientific areas. Although widely acclaimed as “the father of modern chemistry” in standard histories of chemistry [10,13], revisionists correctly identify that he was not alone in the chemical firmament, but disagree about the degree of influence that he had on the development of the subject [64,65,66]. Fortunately, it is not our mandate to enter into this debate. Even as a young man, Boyle was a convert to atomistic or corpuscularian views, and an early work dating from 1654 (only published after his death) entitled *Atomicall Philosophy* is essentially a restating of the views of Gassendi, Descartes, and Sennert [67,68]. However, Boyle’s 1661 publication *The Sceptical Chymist*: or *Chymico-Physical Doubts & Paradoxes* [69] is of relevance to our story. This monograph is written as a discourse on the nature of matter between five protagonists and which Boyle uses to forward the atomist (or rather, corpuscularian) philosophy. The text is rather difficult for the modern reader, who is generally not accustomed to scientific arguments being presented in a discursive style, but this would have been familiar to his contemporary scientists and philosophers. Although Boyle was probably not the originator of the modern definition of an element, he discarded the Aristotelian four elements for a more robust and experimentally verifiable understanding of an element:

“I now mean by Elements, as those Chymists that speak plainest do by their Principles, certain Primitive and Simple, or perfectly unmingled bodies; which not being made of any other bodies, or of one another, are the Ingredients of all those call’d perfectly mixt Bodies are immediately compounded, and into which they are ultimately resolved”.

However, Boyle the experimentalist did not believe that any of the materials known to science at that time were “perfectly unmingled bodies” (elements), although there is a relatively long list of substances known to him that we today call elements, including antimony, arsenic, bismuth, carbon, copper, gold, iron, lead, mercury, silver, sulfur, tin, and zinc. We have already noted that Boyle was an early adopter of the term *molecula* to describe an assembly of atoms or corpuscles. Boyle proposed that chemical change was associated with rearrangements within or between clusters of corpuscles—a concept that can be correlated with the modern view of chemical change being associated with the making and breaking of bonds. In another of his works, *The origine of formes and qualities*, Boyle gives a description of the assembly of the corpuscles that resonates remarkably with the modern molecular model [70]:

“There are also Multitudes of Corpuscles, which are made up of the Coalition of several of the former Minima Naturalia; and whose bulk is so small, and their Adhesion so close and strict, that each of these little Primitive Concretions or Clusters (if I may so call them) of Particles is singly below the discernment of Sense …”

Although Boyle was a chemist from the Age of Reason *par excellence*, he apparently retained a foot in the alchemical roots of his subject. The debate continues as to where his heart really lay, on the one hand he is seen as the alchemist criticizing some alchemical theories rather than defending a modern view of chemistry [71] and who went as far as communicating to the Royal Society his conversion of mercury into an “essential mercury”, which would allow transmutation to gold [72], and on the other he is eulogized as the first true scientist [73].

#### 2.4.3. Isaac Newton—Another Alchemist?

Sir Isaac Newton addressed the question of attraction between atoms in Query 31 of his work *Opticks* [74], and gives a good contemporary view of the ideas concerning the forces between atoms. After writing “The Parts of all homogeneal hard Bodies which fully touch one another, stick together very strongly. And for explaining how this may be, some have invented hooked Atoms, which is begging the Question; and others tell us that Bodies are glued together by rest, that is, by an occult Quality, or rather by nothing; and others, that they stick together by conspiring Motions, that is, by relative rest amongst themselves”, he continues with his own ideas, “I had rather infer from their Cohesion, that their Particles attract one another by some Force, which in immediate Contact is exceeding strong, at small distances performs the chymical Operations above-mention’d, and reaches not far from the Particles with any sensible Effect”. In the text, Newton shows a broad knowledge of chemistry but, like most of his contemporaries, does not always make a clear distinction between physical change and chemical reaction. In many respects the ideas of Newton are closely related to the ideas of chemical affinity and he gives examples of chemical reactions arranged in a similar manner to Geoffroy (see Section 3.1.1).

#### 2.4.4. Bryan and William Higgins—A Historical Aside

Another player in our unfolding drama is Bryan Higgins (1737?–1818) who practiced as a physician and published a number of treatises and books on practical chemistry. He was interested in the forces present in (classical) elements and developed a model based upon the repulsion of like atoms in all elements except earth and water [75]. In this model, he further developed Newton’s ideas of mutual repulsion between atoms (or molecules in modern terms) of gases. He also developed a model for the formation of compounds AB from acids and bases (in his terminology, *atoms* of A and B) based upon a greater attractive force in AB than the repulsive forces between A and B atoms. This predates the Brönsted acid-base model and anticipates ionic bonding between cations and anions.

William Higgins (1762?–1825) was the nephew of Bryan Higgins and shared the familial interest in the attractive forces between atoms and refined his uncle’s model to consider interactions between more than two particles [76,77]. The similarity of the vector diagrams of the forces that Higgins presents to modern covalent bonds is remarked upon by Partington [11].

## 3. The Nineteenth Century C.E.

### 3.1. Valence and Affinity

By the beginning of the 19th century C.E., the scientific world was developing a large enough body of chemical knowledge to begin meaningful discourse on the forces holding atoms together in molecules. One of the critical aspects was the development of a theory to explain and predict how many and what type of atoms could be bonded to each other. The time has come for a discussion of valence or valency.

#### 3.1.1. Chemical Affinity—Driving Force but not Bonding

**Figure 5:**
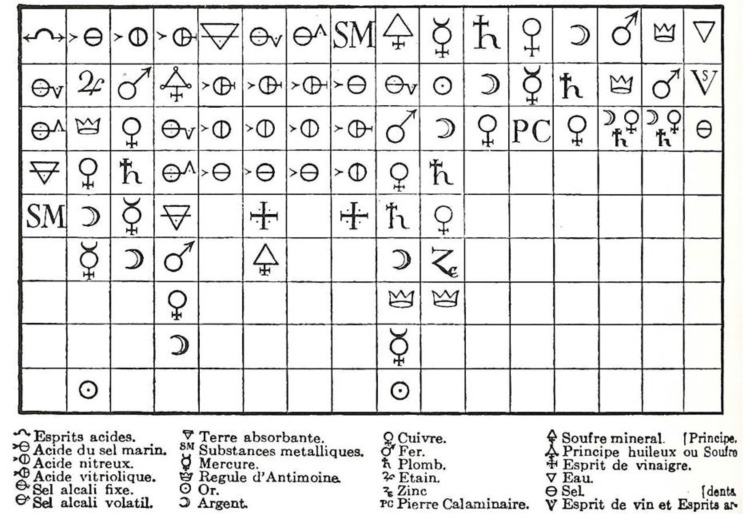
Geoffroy’s affinity tables represented one of the early attempt to bring order to the wealth of chemical information [84,85,86] (Image source, Public Domain, https://commons.wikimedia.org/wiki/File:Affinity-table.jpg).

By the 19th century C.E., the term affinity was understood to refer to the number of, and preference for, the different combinations into which substances could enter and form new compounds. The term had been used earlier with some subtly different meanings [78,79]. One early usage is attributed to Albertus Magnus (1193–1280) who used the word *affinitas* in the context of chemical reactivity, for example he describes the reaction of sulfur with metals on the basis of a shared *affinitas* [80,81,82]. In general, Magnus expounded the Aristotelian vision and although his use of affinity is very early, it has little further relevance for us. The same applies to contributions on chemical affinity or elective affinity from Francis Bacon, Robert Boyle, John Mayow, Johann Rudolf Glauber, Nicolas Lémery, Isaac Newton, and Georg Ernst Stahl [83]. In general, the concept of affinity evolved into those of driving force, free energy, and chemical thermodynamics. Nevertheless, it is worth noting that Étienne François Geoffroy (1672–1731) [84,85,86] and Torbern Olof Bergman [87,88] both produced “affinity tables”, which ordered substances according to their tendency to react with each other (Figure 5).

The difficult relationship between chemical affinity and valence was well expressed by Falk in 1914, when he wrote, “The separation of these two problems, valence and chemical affinity, makes it clear that while a great number of substances may be predicted from a consideration of valence structures alone, questions of chemical affinity, or relative stability, limits the number of these substances which are actually known or may be prepared” [89].

#### 3.1.2. Valence or Valency

For a true understanding of modern chemistry, the concept of valence (or valency) is critical [90,91]. The IUPAC definition, “The maximum number of univalent atoms (originally hydrogen or chlorine atoms) that may combine with an atom of the element under consideration, or with a fragment, or for which an atom of this element can be substituted” is unambiguous and, thus, assigns valences of four and five to carbon in methane CH_4_ and methanium CH_5_^+^, respectively. The principal driver for the acceptance of the modern sense of valence was August Kekulé [92]. In one of his critical publications, Kekulé wrote “*Die Zahl der mit Einem Atom (eines Elementes, oder wenn man bei zusammengesetzteren Körpern die Betrachtung nicht bis auf die Elemente selbst zuruckfuhren will, eines Radicales) verbundenen Atome anderer Elemente (oder Radicale) ist abhängig von der Basicität oder Verwandtschaftsgrösse der Bestandtheile*” (The number of atoms of other elements (or radicals) connected with an atom (an element, or, if one does not want to reduce the observation to the elements themselves in the case of composite bodies, a radical) depends on the basicity or relationship size of the components) [92]. Valence was critical as a driver for the understanding of organic chemistry and also the development of the coordination chemistry model proposed by Alfred Werner. Nevertheless, it is a difficult and diffuse concept and Ramsberg comments, “Strictly speaking, valence … was a number that possessed no physical significance” [90]. The frustration in the 19th century C.E. was tangible: in 1876, Victor Meyer wrote, “*Allein diese Arbeitsfülle und die grosse Zahl der gewonnenen Vortheile haben niemals das Bewusststein unterdrücken können, dass wir über das eigentliche Grundprincip unserer heutigen Anschauungen, über die Nature dessen, was wir eine Valenz oder Verwandtschaftseinheit nennen, vorläufig noch vollkommen im Unklaren sind*” (The sheer volume of work and the large number of advantages gained have never been able to suppress the awareness that we are currently completely unclear about the basic principle of our current views and the nature of what we call valence or affinity) [93].

**Figure 6:**
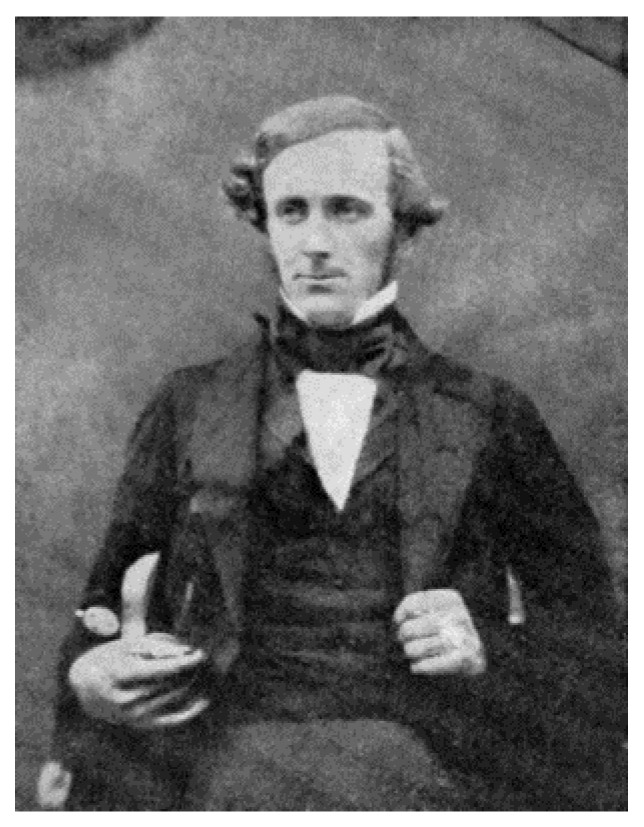
Edward Frankland (1825–1899) was the father of valence—or rather “combining power” as he phrased it. (Public domain image taken from https://en.wikipedia.org/wiki/Edward_Frankland).

The true modern definition of valence originated with Edward Frankland (Figure 6) in 1852 when he observed, “When the formulae of inorganic chemical compounds are considered … it is sufficiently evident … no matter what the character of the uniting atoms may be, the combining power of the attracting element… is always satisfied by the same number of these atoms” [94]. Frankland’s “combining power” is the first formulation of the concept of valence and was adopted by the German community, being translated variously as *Sättigungskapazität*, *Atomigkeit*, *Werthigkeit* and eventually as *Valenz*. It is probably worth quoting Lothar Meyer at length from the first (1864) edition of *Die Modernen Theorien der Chemie und Ihre Bedeutung für die Chemische Statik*, where he wrote:

“The four groups of elements are usually distinguished according to a traditional, but not very well chosen, designation, as *mono-, di-, tri- and tetraatomic* or *mono-, di-, tri- and tetrabasic*. The former expressions are unsuitable because one cannot easily distinguish the usage from *monatomic* etc. *atoms*. The expressions *monobasic* etc. remind us that the doctrine of multiple saturation capacity derives its origin from Liebig’s classical investigations of polybasic organic acids. But since we are used to understand basic as the opposite of acid (in the chemical sense), this expression cannot be applied to the atoms either. However, it does not seem to be easy to replace it with another expression that is both comfortable and appropriate. The most correct and strictest way is probably to describe the atoms as those with 1, 2, 3 and 4 times the saturation capacity. The half-Greek and half-Latin expressions used by Wislicenus are shorter, namely “monaffin, di-, tri- and tetraffin”; in German one could also say “mono-, di-, tri- and quadrivalent”, for which, according to the judgement of tolerant philologists, “uni-, bi-, tri- and quadrivalent” could also be used. Characteristic and for many cases convenient is also the expression, according to which the four groups of atoms are usually described as having 1, 2, 3 and 4 “kinship units”.

[95]

Note that in this translation, -*werthig* has been rendered as -valent and *Verwandschaftseinheiten* as associated units. By 1872, in the second edition of his book, Lothar Meyer was widely using the terms *der chemische Werth* and *die Valenz* [96]. This use of Valenz seems to be due to Carl Wichelhaus [97] who wrote “*Gebraucht man „Valenz” als kürzeres Wort an Stelle des von A. W. Hofmann eingeführten „Quantivalenz” in demselben Sinne, so ist es zunächst klar, dass zur Bestimmung der relativen Grösse dieser „atomfesselnden Kraft” nur diejenigen Verbindungen dienen können, welche ein Molecul repräsentiren*” (If one uses “valence” as a shorter word in place of the “quantivalence” introduced by A. W. Hofmann in the same sense, it is initially clear that only those compounds which represent a molecule can serve to determine the relative magnitude of this “atomic bounding force).

Another problem that confronted the early workers was that of variable valence. In 1852, Frankland recognized that some elements could have multiple valences [94]. For example, the group 15 elements formed compounds with combining power (valence) of three, such as NH_3_, PH_3_, and PCl_3_, but also with a combining power of five, as in NH_4_I and PH_4_I. Note that in these representations, no distinction is drawn between the number of atoms bonded to the group 15 atom (four) and the total number of additional atoms (five), as the ionic nature of these compounds was not explicitly recognized by Frankland. Frankland does not appear to have compared PCl_3_ and PCl_5_, although both had been prepared earlier in the century [98,99].

**Figure 7:**
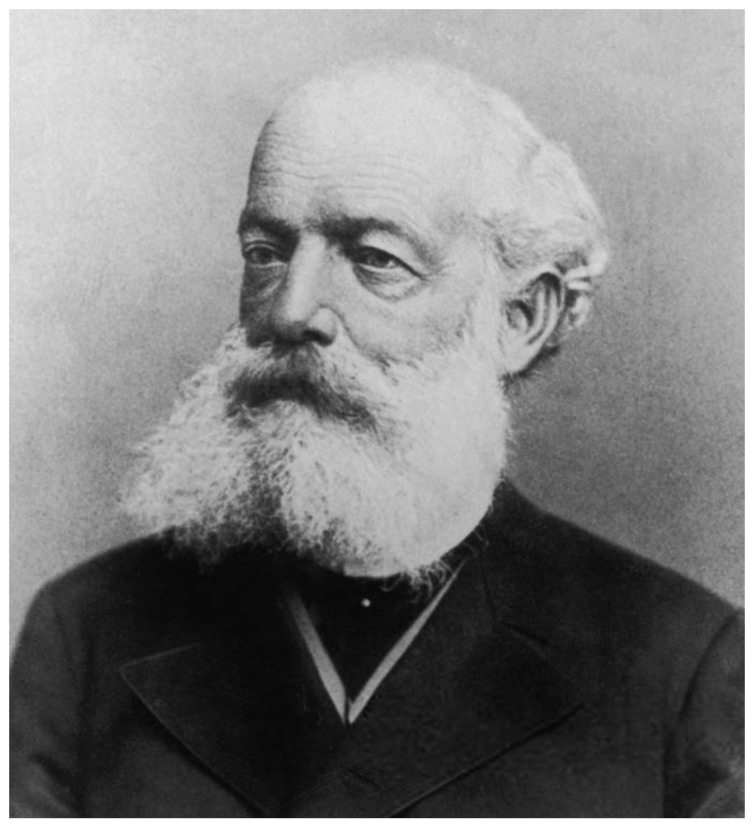
Friedrich August Kekulé (1829–1896) embraced the valence model and used it to rationalize organic chemistry (Public domain image taken from https://en.wikipedia.org/wiki/August_Kekulé/media/File:Frkekulé.jpg).

August Kekulé (Figure 7) proposed that elements had a fixed valence but, although he was aware of the work of Frankland, he did not recognize that combining power was the same as his valence [100]. This model of fixed valence was very successful in rationalizing the chemistry and the structures of organic compounds using a fixed valence of four for carbon, and allowed the extension to elements such as nitrogen and oxygen, which were proposed to have fixed valences of three and two, respectively. One of the successes of the model was that the fixed valence of four for carbon necessitated the presence of multiple bonds (or free valences). An early publication relating to chemical topology and graph theory was predicated upon the fixed-valence model for carbon, although the author, Oliver Lodge from University College London had, apparently, no great love for the word valency, and wrote, “Might I suggest the term “order” for non-chemical use, instead of atomicity or valency, which, though doubtless they do very well in chemistry, are not pleasant words? The change of zero might be made at the same time, and atoms of tetravalent atomicity be called atoms of the 2nd order, and so on” [101].

Even Kekulé had to admit to reality and accept that some compounds did not fit well in his universal view and he introduced the description *“molecular compounds”* for substances that did not conform to his valence rules; “*A côté de ces combinaisons atomiques nous devons distinguer une seconde catégorie de combinaisons, que je désignerai par le nom combinaisons moléculaires”* (In addition to these atomic combinations we must distinguish a second category of combinations, which I will refer to as molecular combinations) [102]. Taking PCl_5_ as an example, Kekulé re-formulated the compound as PCl^3^, Cl^2^ which evolved into the more modern dot notation PCl_3_⋅Cl_2_.

By the latter part of the 19th century C.E., valence or one of its synonyms was sometimes being used in the sense oxidation number and sometimes to refer to the number of bonded atoms. The situation was well-summarized by Madan in 1869, “It is very much to be regretted that the subject of chemical nomenclature is in such an unsettled state. It seems a real reproach to chemists that scarcely two text-books can be found in which the same system of names is adopted, and that there is hardly a single number of a scientific periodical which does not contain specimens of totally different systems” [103].

### 3.2. Dalton and the Full Glory of Atomism

**Figure 8:**
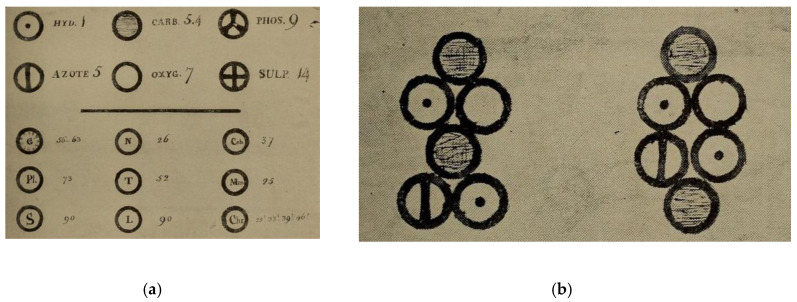
(**a**) John Dalton introduced atomic symbols as a part of his atomic model and (**b**) anticipated the occurrence of isomers in which atoms were arranged in different spatial manners (1829–1896) embraced the valence model and used it to rationalize organic chemistry [113,114,115]. (Image taken from https://www.biodiversitylibrary.org/bibliography/9535).

A crucial step in the development of our understanding of bonding is due to John Dalton (1766–1844). Instead of using alchemical symbols to describe real elements, philosophical elements and compounds [104], he proposed that elements should be denoted by symbols [105,106]. Furthermore, he recognized that these symbols could be combined to give representations of molecules and compounds that clearly identified the number and type of atoms of which they were composed. It is probably true to say that Dalton was responsible for the acceptance of the atomic theory by the broad body of scientists. His ideas were developed in 1803, but only broadly disseminated in his book *A New System of Chemical Philosophy* published in 1808. Dalton arranged his elements in order of atomic weight in a manner that is familiar to the modern chemist, although the atomic weights of the time differ from those we use now (H = 1, O = 7, and N = 5). The differences arise from Dalton’s assumption that, “When only one combination of two bodies can be obtained, it must be presumed to be a binary one, unless some cause appear to the contrary”. This assumption resulted in him formulating water and ammonia as HO and HN, respectively, with the consequence that the equivalent weights for combination with hydrogen were 1/2 and 1/3 of the “real” values. As mentioned earlier, Dalton used the term atom for both atoms and molecules (the atomic weight of ammonia, NH, was 6). The atomic symbols used by Dalton comprised a circle modified to uniquely describe an element. Although these were soon replaced by Jöns Jacob Berzelius with modern alphabetic symbols [107,108,109,110,111], Dalton’s contribution to bonding was to introduce a notation in which substances could be represented by combinations of atomic symbols. This implies a persistent interaction between the atoms—in other words, bonding. It is unclear whether Dalton considered that the representations were a precise representation of the spatial arrangement of atoms in molecules. Dalton clearly identified that spherical atoms of different elements could be different sizes but must have the same weight. In terms of shape, he is ambivalent and criticizes Berzelius’ view that “a compound atom cannot be considered as spherical, but that an elementary atom may be taken as such” [112]. On the one hand he states that he does not “see any reason sufficient for all simple atoms to be” spherical, and continues “those of hydrogen may be spherical perhaps; those of oxygen may be regular tetrahedrons; those of azote may be cylinders of equal diameter and altitude; &c, &c” [112]. Regarding compounds, his view is that, “Of all compound atoms, that consisting of 3 elementary atoms is probably most remote from a sphere, but when one compound contains 5 or more simple ones, the figure must, I should suppose, be virtually a sphere” [112]. Whether or not Dalton was convinced about the reality of the spatial arrangement, he clearly identified that the different arrangement of the atoms could define different compounds, using different arrangements of the atoms in the formula C_2_H_2_ON to describe albumen and gelatin (Figure 8) [113,114,115].

### 3.3. Isomerism … the Agony of Being the Same but Different

By the beginning of the 19th century C.E., chemists generally had an understanding that a single chemical formula represented a single compound, in part an unjustified extension of Proust’s law [116]. This was a view at odds with that expressed by Henry Cavendish before the nomenclature reforms of Lavoisier; Cavendish stated in 1787 that he thought it “very wrong to attempt to give [compounds] names expressive of their composition” [117]. This comfortable state of affairs was soon to end [118]. With the notation of Dalton and Berzelius in place, the constitution of compounds could be expressed by the combination of atomic symbols. In 1824 Liebig and Gay-Lussac described the preparation and properties of silver fulminate, Ag(CNO), which was a compound with interesting properties, “*Le fulminate d’argent ne détone jamais seul à la température de 100°, ni à celle de 130°; mais il faut éviter de l’exposer au plus léger choc entre deux corps durs, même lorsqu’il est dans l’eau*” (Silver fulminate never detonates alone at the temperature of 100° or 130°, but it should not be exposed to the slightest shock between two hard bodies, even when it is in water) [119]. In the same year, Friedrich Wöhler described silver cyanate (*cyansaure Silberoxyd*) and reported the analysis Ag(OCN)—in other words, the identical formulation to Liebig’s silver fulminate [120]. After a few exchanges between Liebig [121] and Wöhler [122], the former eventually conceded that the two compounds had an identical formulation but different properties [123]. Not content with upsetting the inorganic community, in 1828 Friedrich Wöhler proceded to cast confusion and shock into the organic and philosophical communities [124]. In purely chemical terms, Wöhler showed that syntheses designed to yield ammonium cyanate, [NH_4_](OCN), gave the isomeric organic compound, urea OC(NH_2_)_2_ [125,126,127,128]. The philosophical challenge related to the doctrine of vitalism, which maintained that organic compounds possessed special properties because they contained a vital force as a result of being formed by living things [129,130,131]. Although the Wöhler urea synthesis was certainly one of the experiments that resulted in the questioning of the vitalism theory, it is now clear that it was by no means responsible for its discrediting, although this revision has not yet made its way to the chemical text-books [132,133,134]. The difference between silver fulminate and silver cyanate, with the modern formulations Ag(CNO) and Ag(OCN), respectively, leads irrevocably to the need for chemical bonding and the consequence that atoms have fixed interactions with specific other atoms and ultimately that the bonding of atoms implies a three-dimensional arrangement in space.

But we get a little bit ahead of the story. It took Berzelius to make the necessary leap of imagination. Berzelius had an array of disparate results available to him, which his genius correlated into one of the most important insights in the development of modern chemistry. Amongst these results were the cyanate/fulminate and ammonium cyanate/urea conundrums and in 1830, in a paper entitled *Ueber die Zusammensetzung der Weinsäure und Traubensäure (John’s Säure aus den Vogesen), über das Atomengewicht des Bleioxyds, nebst allgemeinen Bemerkungen über solche Körper, die gleiche Zusammensetzung, aber ungleiche Eigenschaften besitzen*, Berzelius proposed that these pairs of compounds should be described as homosynthetic or isomeric (Greek, *ἰσομερής*, equal part) bodies and favored the latter term [135].

### 3.4. Organic Chemistry and Structure—The Bond Reigns Supreme

After the insights of Berzelius, the stage was set for the growth of organic chemistry into a rigid intellectual discipline [136]. This is, unfortunately, not the place for a detailed discussion of the fascinating history of the representation of bonds in the development of organic chemistry [137,138,139,140,141].

The honor of introducing the first new type of bond probably goes to Alexander Butlerov (1826–1888) when he incorporated the double bond into organic structures. In doing this, he was also one of the pioneers of representing organic molecules with a two-dimensional structure connected by lines, representing the bonds or valences [142]. As Butlerov wrote, “Starting from the assumption that each chemical atom possesses only a definite and limited amount of chemical force (affinity) with which it takes part in forming a compound, I might call this chemical arrangement, or the type and manner of the mutual binding of the atoms in a compound substance, by the name of chemical structure” [143,144,145]. This can be seen as the beginning of the structural theory of organic chemistry [146]. An excellent overview of the competing claims and counter-claims for the discovery of structural formulae, the incorporation of multiple bonds and the genesis of the tetrahedral carbon atom has been recently published by Rocke [147].

Amid the claims and counter-claims, it is worthwhile seeing how bonds were interpreted in the general organic chemistry community at this time. Although the bonding had a topological meaning in terms of how the atoms were connected as the structural theory developed, the general understanding of chemists was that the depictions which correctly showed the connectivity, did not imply anything regarding the positions of the atoms in space [139,148]. For us, it is convenient to start the story with Archibald Scott Couper (1831–1892) [149,150,151] and August Kekulé [100] who almost simultaneously proposed that tetravalent carbon atoms could link together to form chains with C–C bonds, building on Charles Gerhardt’s ideas about homologous compounds differing by the addition of CH_2_ moieties [152]–and so was modern organic chemistry born! Alexander Crum Brown (1838–1922) had introduced his croquet-ball notation (which persists to this day with the convention of white, red, black, and blue colorations for hydrogen, oxygen, carbon, and nitrogen atoms, respectively) for representing chemical structures in 1864 [31,153,154]. In these structures, the four lines to a carbon atom represent the valency of four and do not imply any specific spatial structure. Crum Brown and Frankland were at pains to emphasize this point. Hein stated, Kekulé’s theories “required no specific arrangement in space but did refer to chemical relations between atoms in three dimensions [155]. However, as the 1860s developed, Kekulé was shifting his ideas more and more to atoms having a distinct spatial as well as valence relationship. In the models he used to represent molecules, a saturated carbon atom was represented by a tetrahedron. By 1865, Kekulé had proposed the hexagonal structure for benzene and had also identified different isomers of the disubstituted derivatives [156,157].

Although Kekulé had perhaps opened the floor for the discussion of the three-dimensional arrangement of molecules, it was left to Jacobus Henricus van’t Hoff (1852–1911) [158,159,160] and Joseph Achille Le Bel (1847–1930) [161] to independently demonstrate that a three-dimensional, tetrahedral carbon atom was needed to explain aspects of stereochemistry related to stereogenic centers and chiral molecules. In particular, van’t Hoff used his ideas to explain the stereochemical consequences of cumulated double bonds in allenes and cumulenes [162]. Although the new ideas were not immediately accepted, their popularization and championing by Johannes Wislicenus (1835–1902) resulted in their eventual acceptance [163,164].

From the middle of the 19th century C.E., bonds were routinely being represented with the lines that we are familiar with today, although it is also fair to say that there was little understanding or consensus as to what the lines actually meant. The prevalence of the Kekulé view and its emphasis on a fixed valence of four for carbon was instrumental in this development. One debate, which is again beyond the scope of this article, was related to the question of whether the depiction of the bonds implied anything about the spatial arrangement of the atoms. Slowly the organic chemistry community came to accept this, with the critical observations relating to isomerization and the need for a fixed three-dimensional structure. An excellent account is given in Ramberg’s book *Chemical Structure, Spatial Arrangement. The Early History of Stereochemistry, 1874–1914* [90].

### 3.5. Strings and Things—Jørgensen and Coordination Chemistry

The next part of our story begins in 1839, with Charles Gerhardt (1816–1856) introducing his model of organic chemistry, which consisted of the making and breaking of bonds between residues. In reality, Gerhardt’s residues were closely related to radicals. He originally called the process of joining the residues together copulation, but later used the description “double decomposition” [165,166,167,168,169,170,171,172]. Although Gerhardt originally used this for describing salts of organic acids, the model was then further extended, in particular by Jakob Berzelius to cover a more general linkage in a pairwise manner or into chains of copulated compounds (*Paarlinge oder gepaarte Verbindungen*) [173]. This developed most successfully in the organic arena to the chains of carbon at the core of the structural theory that we discussed in the previous sections.

However, we now see another of the deviations from narrative linearity that litters the course of chemical history with the attempts to extend the chain model to inorganic chemistry, in particular to coordination compounds. Berzelius used his annual reviews of progress in science to extend the copulation model and its C–C bonds which rationalized organic chemistry to N–N bonds in metal complexes, denoting [Ni(NH_3_)_6_]Cl_2_ as NiCl + 3NH^3^ in which the strike-through means that the ammonia is copulated in a chain (note the atomic and equivalent weights used by Berzelius gave the formula quoted and also Berzelius’ use of raised integers to denote composition [174]. This approach was initially embraced by the inorganic community as it provided some structure to the study of the poorly understood coordination compounds. It fell to Blomstrand to further develop and expand the Berzelius ideas and in *Die chemie der Jetztzeit vom standpunkte der electrochemischen Auffassung. In Aus Berzellius Lehre entwickelt,* he introduced a new notation for the copulated ammonia molecules and extended the system to other ligands such as cyanide in K_4_[Fe(CN)_6_] [175]. Although an interesting historical aside, and not so fanciful as they initially appear to our modern eyes, these structural representations evolved in the absence of any knowledge of what a bond might actually be and before the electron had been discovered. Nevertheless, the model had the major deficit that it was unable to predict how many of a particular ligand might be associated with a particular metal salt.

The Blomstrand model was adopted by Sophus Mads Jorgensen and used to rationalize a vast body of experimental work and precise observations reasonably well [176,177,178]. Jorgensen accounted for the valence in compounds such as [Co(NH_3_)_6_]Cl_3_ by proposing three separated chains of ammonia ligands each terminated with a chlorine [179]. There remained three difficulties which were not addressed: (i) the nature of the interactions between the ligands and or atoms in the conjugated chain were unknown, (ii) there was no rationale what molecules could act as ligands, and (iii) at the time that organic chemistry was developing an awareness of the third dimension, this was neglected in the coordination compounds.

### 3.6. Primary and Secondary Valences—Or When is a Bond not a Bond?

The Jörgensen ideas were eventually replaced by Werner’s coordination model. It is not appropriate to rehearse the controversy between these two scientists in this article [180,181,182], but we should note that Werner was influenced by the developing three-dimensional understanding of organic chemistry and developed a new way of rationalizing the structure of coordination compounds. In 1893, Alfred Werner proposed his model in *Beitrag zur Konstitution anorganische Verbindungen* [183]. He surveyed the literature of coordination compounds, in particular the work of Jørgensen, and rationalized the compounds in terms of a primary valence (*Hauptvalenz*) and a secondary valence (*Nebenvalenz*). These are equivalent to oxidation state and coordination number, respectively. This topic has been discussed in detail elsewhere [184]. Now that the detour into chain theory of inorganic compounds has been corrected, we can return to our main theme and the birth of the modern understanding of bonds and bonding.

## 4. The Modern Era

### 4.1. The Advent of the Electron and Ions

Electricity has a long and noble history, with empirical observations dating back to the ancient civilizations of Egypt and Greece [185,186,187,188,189,190,191]. This is not the place to relive these early experiences, ranging from electric shocks from fish and reports on the phenomenon of static electricity, nor is it appropriate to enter into the debate regarding the function of what is claimed to be a galvanic cell dating to Parthian era (250 B.C.E.–230 C.E.) [192,193,194]. For us, the story of the electron properly starts in 1600, when William Gilbert [195,196,197] published his work on magnets and magnetism [198]. In this book, Gilbert serendipitously connects magnetism and electricity as he distinguishes between the magnetic effect, typified by the behavior of lodestone, and static electricity as exhibited by substances such as amber. To Gilbert, we owe all the modern *electr*- words, including electric, electricity and electron; Gilbert derived the word *electricus* from the Latin *electrum* and Greek ἤλεκτρον (elektron) meaning amber and used it adjectivally in *de Magnete*, “*Vim illam electricam nobis placet appellare*”. (We prefer to call it electric power). As early as 1620, Francis Bacon devoted a section of his *Instauration Magna* Part III: The Phenomena of the Universe; or a Natural and Experimental History for the foundation of Philosophy, to electrical phenomena (Electricity: 1. The bodies that are electrical; 2. The bodies that are not electrical; 3. The bodies disposed to be attracted; 4. Leading experiments made with electrical bodies) [199].

The recognition that the electrical phenomenon was associated with two different types of effect emerged in the course of the 18th and 19th centuries C.E. [200,201,202], commencing with the observation by Charles François de Cisternay du Fay (1698–1739) that silk and amber had opposite attraction or repulsion properties, and interpreted this is in terms of their possessing different fluids [203,204]. The transition from a fluidic vision of electricity to one involving charges was made by Benjamin Franklin in 1747, when he introduced the terms positive and negative [205]. These terms still related to a fluidic model and referred to and excess or a deficit of an electrical fluid. Thus, began a debate about the direction of flow of electrical charge that mystifies students to this day. In the United States, Ebenezer Kinnersley [206] made similar observations to du Fay, describing them in a 1762 letter to Benjamin Franklin in which he uses the latter’s negative and positive notation [207].

The first association of the electrical fluid with atomic and molecular structure seems to stem from the work of Richard Laming [208,209,210,211]. He developed a model composed of central atoms surrounded by electrical particles in which “different sorts of atoms are naturally associated with unequal quantities of electricity”—rephrase this as “atoms of different elements are associated with different numbers of electrons” and you have a very modern view of atomic structure [211]. Laming considered atoms to be solid, a view that was contested by Sloggett, who otherwise had a very similar vision of atomic structure [212]. As the 19th century C.E. progressed, the concept of a charged particle that behaved like an atom of electricity became established [200,213], and it fell to George Johnstone Stoney to propose the name electrine for this particle in 1881 [214]. Stoney was also one of the first (if not the first) to recognize that there was a fundamental unit of electricity and his work seems to have predated that of Hermann Ludwig Ferdinand von Helmholtz [215]. Although first published in 1881 [214,216], the text in which Stoney proposes a fundamental unit of electricity is that of a lecture presented in 1874 [217].

We now come to the electron [202]. The name electrine, proposed in 1881 [214,216] was widely ignored by the community and Stoney modified this to electron in 1894 [213]. The Helmholtz memorial lecture given by George Francis Fitzgerald is remarkable for a number of reasons [218]. Fitzgerald talks extensively about electrons, but nevertheless writes, “The suggestion that electrons have an individual existence is undoubtedly tempting, but it is worth while keeping constantly in view the possibility that their constancy of quantity is connected with a constancy of structure of the ether rather than with any individual existence of each electron” [218]. Like Helmholtz, he relates the electron to chemical bonding, but once again, there is a conflict with modern electrostatic ideas when he writes, “This follows from considering that the work done in the combination of H and Cl may be mostly due to the attraction of electrons” [218].

**Figure 9:**
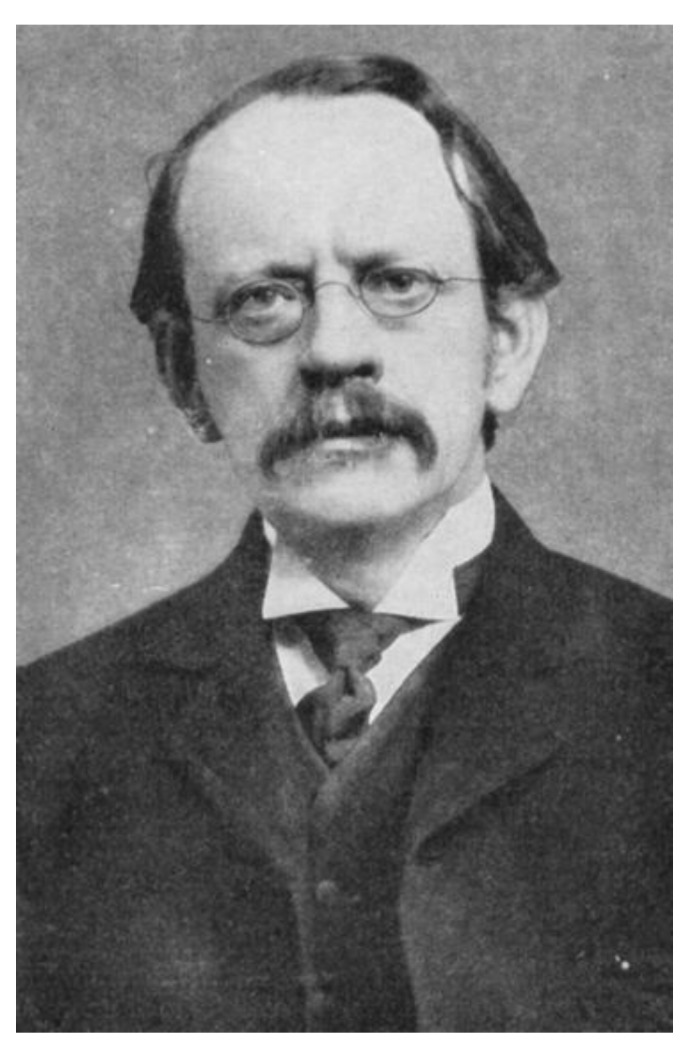
Sir Joseph John Thomson (1856–1940), the discoverer of the electron. Once the electron had been discovered, the time was ripe for modern theories of bonding to emerge. (Public domain image taken from https://en.wikipedia.org/wiki/J._J._Thomson#/media/File:J.J_Thomson.jpg).

One year after Fitzgerald cast doubts about the individual existence of the electron, Joseph John Thomson (Figure 9) reported their experimental observation [219]! Thomson was studying the emanations of the cathode ray tube, originally described by William Crookes, and performed experiments indicating that cathode rays really were unique particles. At the time, a debate was raging as to whether the rays were waves, atoms, or molecules, but Thomson made the case for cathode ray particles and provided estimates of their charge and mass, the latter being very much smaller than that of the lightest known atom, hydrogen. Thomson described the phenomenon and the new corpuscles thus: “If, in the very intense electric field in the neighbourhood of the cathode, the molecules of the gas are dissociated and are split up, not into the ordinary chemical atoms, but into these primordial atoms, which we shall for brevity call corpuscles; and if these corpuscles are charged with electricity and projected from the cathode by the electric field, they would behave exactly like the cathode rays” [219]. An excellent introduction to the scientific background and the prevalent scientific thought prior to his discovery is presented in Thomson’s Nobel Prize address from 1906 [220]. By 1908, the electron was sufficiently established as a reality in chemistry, that William Ramsay could give a lecture entitled *The Electron as an Element* as his presidential address to the Royal Society of Chemistry [221].

The discovery of the electron and its importance in the understanding of chemical bonding cannot be over-emphasized. The history of chemical bonding can with justification divided into a pre-history before the electron and the modern era post-1897.

### 4.2. Ionic Bonding

Now that the electron has entered our vocabulary, we can move directly to a discussion of bonding in modern terms. We will jump over the discovery of the proton and the neutron, together with the development of the atomic structure model with a small central nucleus surrounded by electrons [222,223,224,225,226]. These are critical developments in the history of modern chemistry, but only tangential to our main theme of bonding, which is to do with electrons rather than nuclei. Indeed, it is fair to say that most historical studies of atomic structure completely ignore the issue of bonding.

Ionic bonding fits perfectly within the IUPAC definition of bonding [1], although it might cause conceptual difficulties for those embedded within the covalent world of shared electrons. The ionic bond is defined by the electrostatic interaction between negatively and positively charged chemical species. The latter are, in turn, defined by the transfer of an electron or electrons from one atom to another. It is worth making a comment here that “pure” ionic bonding is an ideal concept: in reality, there will always be a finite, but often vanishingly small, probability of finding the “transferred” electron or electrons close to their mother atom. In other words, there will always be a degree of covalency. We discuss this aspect later.

Ions had become familiar to chemists in the course of the 19th century C.E. Michael Faraday first demonstrated that solutions of certain compounds in water could conduct electricity. He proposed that the action of the electricity caused the compounds to break up into charged particles which were responsible for the conductivity. In his early experiments, he does appear to have considered that the ions might be present in the parent compound itself. Farady also introduced the new term ion (Greek, wanderer) and recognizing that the ions had charges, also introduced the descriptions cation and anion.

It was another half century before Svante August Arrhenius made the important proposal that the charge carrying species, the ions, could be atoms carrying a positive or negative charge. It was also Arrhenius who recognized that formation of the cations and anions in aqueous solution was a result of the dissolution itself and not, as Faraday had proposed, through the action of electricity.

The discovery of the electron by Thomson was critical to the understanding of ions and ionic bonding and Thomson went as far as suggesting that in a compound like HCl, the atoms were joined by an electromagnetic force, “There seems to me to be some evidence that the charges carried by the corpuscles in the atom are large compared with those carried by the ions of an electrolyte. In the molecule of HCl, for example, I picture the components of the hydrogen atoms as held together by a great number of tubes of electrostatic force; the components of the chlorine atom are similarly held together, while only one stray tube binds the hydrogen atom to the chlorine atom”; he also proposed that HCl had a permanent dipole, with positive and negative ends [219]. Thomson returned to this theme in his 1904 book *Electricity and Matter*, when he wrote, “If we interpret the ‘bond’ of the chemists as indicating a unit Faraday tube, connecting charged atoms in the molecule, the structural formulae of the chemist can be at once translated into the electrical theory. . . but the symbol indicating a bond on the chemical theory is not regarded as having direction, no difference is made on this theory between one end of the bond and the other. On the electrical theory, however, there is a difference between the ends, as one corresponds to a positive, the other to a negative charge” [227]. Interestingly, the word bond only occurs on a very few occasions in this book, but it is clear that he is now talking explicitly about bonding in ionic terms. In the example of ethane, Thomson envisages negatively-charged hydrogen and positively-charged carbon atoms linked by “Faraday tubes”. After starting the discussion of ethane with a model equivalent to [(C_2_)^6+^(H^−^)_6_] where each carbon bears a 3+ charge, he then attempts to rationalize the C–C interaction in terms of Faraday tubes (electrostatic interactions) between these two atoms, resulting in a chemical inequivalency of the atoms, one bearing a 4+ charge and the other 2+. Thus, the framework was in place for an understanding of ionic bonding, or as it was described at the time “polar bonding”, in the first decade of the 20th century C.E. The model was proposed as a universal one for all compounds, even those that did not form ions in solution. Although some of Thomson’s proposals have more to do with covalent bonding, they serve to remind us that from the earliest period, chemists were aware of the dichotomy and inherent difficulties asociated with the distribution of electrons in bonds and in molecules. It took Linus Pauling another 30 years in the future, to introduce the concept of electronegativity.

**Figure 10:**
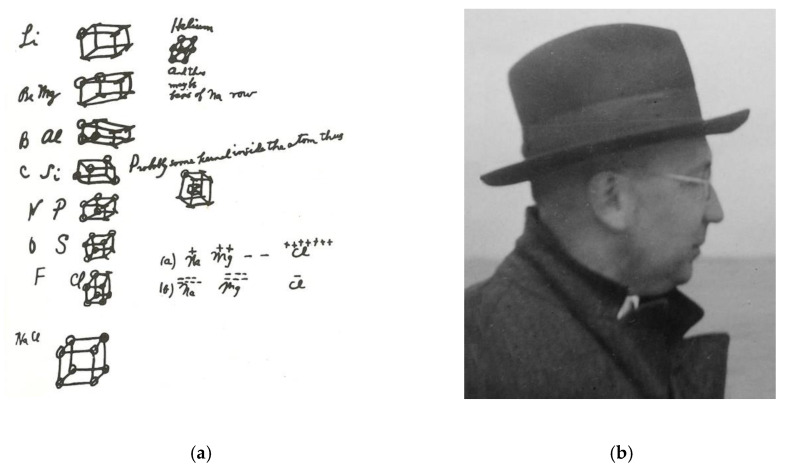
The credit for the development of the modern conceptual models of bonding goes to (**a**) Gilbert Newton Lewis (1875–1946) who placed electrons to be shared at the vertices of a cube (thus conforming to the octet model) and (**b**) Walther Ludwig Julius Kossel (1888–1956) who considered the complete transfer of electrons in ionic bonds. (Images taken from https://en.wikipedia.org/wiki/Gilbert_N._Lewis#/media/File:Lewis-cubic-notes.jpg and https://en.wikipedia.org/wiki/Walther_Kossel#/media/File:Kossel,Walther_1928.jpg).

Another beginning for the modern concept of ionic bonding lies with Gilbert N. Lewis (Figure 10a), whose earliest published work on bonding was in 1913 [228]. However, in 1923 Lewis stated that he made the first of his models based upon the distribution of up to eight electrons to the vertices of a cube in 1902. In papers of 1913 [228] and 1916 [229], Lewis gave a picture of the ionic bond similar in many respects to that of Thomson. Lewis recognized that a stable octet could result either from the sharing of electrons or by the transfer of electrons from one atom to another. He represented the arrangement of eight electrons (the octet) at the vertices of a cube. In his representation, atoms of sodium and chlorine have, respectively, one and seven electrons at these vertices. He postulates the stability of full or empty octets, which can be achieved by the transfer of one electron from the sodium atom (generating an empty octet) to the chlorine (generating a full octet) and building ionic sodium chloride.

As Lewis was developing his ideas in the United States and Thomson was working in the United Kingdom, Walther Kossel (Figure 10b) was following a similar direction in Germany. In 1916, he also identified the special stability of the closed-shell configuration of the elements of group 18 and drew attention to the more general relationships of these closed-shell configurations to those of stable ions [230]. In this paper, Kossel also identifies the positive and negative valencies that can be associated with a particular atom, for example carbon with four valence electrons could lose four electrons to give C^4+^ or gain four electrons to generate C^4−^, both of which are closed-shell configurations “*Jedes Element besitzt sowohl eine positive wie eine negative Maximalvalenz, die sich stets zur Zahl 8 summieren, und zwar entspricht die erstere der Gruppennummer*” (Each element has both a positive and a negative maximum valence, which always add up to the number 8, the former corresponding to the group number) [230]. In this remarkable paper, Kossel, like Thomson and Lewis also identifies the importance of the octet in compounds that we would today describes as covalent (H_2_O, CO_2_, NH_3_, HBr, and CH_4_ for example). These themes were further developed and expanded by Kossel from 1919 onwards. In 1919, Kossel recognized that the formation of stable ions was not limited to the attainment of an octet, but in the heavier elements an 18-electron configuration had a similar stability, typified by the sulfide anion S^2–^. In subsequent papers, also published in 1919, Kossel expanded his vision to both covalent and ionic bonds, discussing carbon compounds and stating, “The behavior of carbon in exhibiting a constant valency, previously considered as particularly simple and typical, must be regarded as exceptional when compared with the majority of elements in which the polar character is marked. In general, a distinction must be made between heteropolar and homopolar linkings” [231,232]. Kossel also extended his model to include coordination compounds and addressed the variable valence exhibited in compounds such as PCl_3_ and PCl_5_.

Although the discovery of ionic bonding is usually attributed to Lewis and Kossel, Thomson should be regarded as an important contributor to the ideas and concepts. However, both Thomson and Kossel acknowledge a debt to Richard Abegg who arguably pointed all three in the direction of the octet. In a 1904 paper entitled *Die Valenz und das periodische System. Versuch einer Theorie der Molekularverbindungen* (Valence and the periodic system. An attempt at a theory of molecular compounds) [233], Abegg noted that for a given element, the sum of the absolute value of its maximum negative valence (such as −2 for sulfur in H_2_S and its maximum positive valence (such as +6 for sulfur in H_2_SO_4_) is usually equal to 8. Abegg was using “valence” in the sense of oxidation number and concludes, “The sum of eight for our normal and contravalences then receives the simple meaning of a number that represents the points of attack of the electrons for all atoms, and the group number or positive valence indicates how many of the eight points of attack electrons must hold in order for the substance to appear as an electron neutral element, the ‘positive’ elements need few (1–3), the ‘negative’ many (5–8) electrons” [233]. Abegg returned to the theme on a number of occasions, developing models that paralleled those being expounded by Thomson, Kossel, and Lewis [234,235]. In 1905 he expanded his ideas to a more general valence theory, and most importantly, clearly identified the confusion arising by the parallel usage of valence and affinity “as soon as the sharp distinction between valence and affinity, which seems necessary to us, is made …almost all contradictions are transformed into agreement” [234]. Also in 1905, Abegg addressed the question of higher oxidation, and stated, “Higher levels of connection can only occur here because these elements do not actuate their negative (“normal”) valences, but their numerous positive (“contra”) valences and thus function *eo ipso* basic or, more precisely, positive” [235].

### 4.3. Covalent Bonding—The Legacy of G.N. Lewis

The relationship between the number of electrons in an atom and the types of compounds it forms in combination with other atoms together with its valence was clearly identified by Thomson [236] and others. As early as 1914, Thomson had realized that there were compounds for which his electrostatic ionic model was not appropriate and considered an alternative in which electrons held the atoms together through a sharing, “When the atoms are electrically neutral, i.e., have no excess of positive over negative charge or *vice-versâ*, for each tube of force which passes out of an atom, another must come in; and thus each atom containing *n* corpuscles [electrons] must be the origin of *n* tubes going to other atoms and the termination of *n* tubes coming from other atoms” [237]. This is essentially the description of a covalent bond with one important exception—for Thomson, a bond seems to be associated with a single electron and a double bond results when two tubes are formed. In a 1921 paper, Thomson clearly identifies the importance of the octet and explicitly relates the arrangement of the eight electrons in an atom to a regular octagon; furthermore, in a manner resembling Lewis, he describes the formation of compounds thus: “Whereas all the plane faces of a cube are four-sided, the twisted polyhedron has 8 triangular faces as well as 2 four-sided ones, thus two such polyhedra could be placed so as to have either 2, 3, or 4 corners in common” [236]. In this same paper, he addresses the question of variable valency in compounds such as PCl_3_ and PCl_5_. Thomson’s views on bonding and other chemical matters are best summarized in a series of lectures from 1923 entitled *The Electron in Chemistry* in which, once again chemical bonding is represented by the fusion of polygons through vertices and edges and faces [238,239,240,241].

Covalent bonding, defined in terms of two-electron two-center bonds between atoms, is usually thought to originate in the 1916 publication by Gilbert N. Lewis [229], although the roots are to be found in the earlier 1913 paper [228]. In his 1916 paper, Lewis visualizes the octet by locating electrons at the vertices of a cube centered on the atom and then builds molecules (or rather bonds) through the sharing of vertices, edges or faces between cubes centered on the bonded atoms. He also introduces the classical Lewis dot structures in this same publication. Although the concept was first formulated by Lewis, it did not excite much attention in the chemical community. The real credit for the general acceptance and adoption of the covalent model goes to Irving Langmuir [242,243,244,245,246,247] and in particular his 1919 paper entitled *The Arrangement of Electrons in Atoms and Molecules*. In this 66-page manuscript, Langmuir retells the story of the Lewis description in terms of a number of postulates and also clearly identifies the change from an octet to a stable 18-electron configuration that can occur in the heavier elements. Equally important was the paper *Polarity and Ionization from the Standpoint of the Lewis Theory of Valence* by Wendell Latimer and Worth Rodenbush in which they relate polarity and reactivity to the bond types exemplified in the Lewis model [248]. In his 1923 monograph, Lewis benefits from the recasting by Langmuir and presents a masterful summary of his theory and also establishes that his original ideas, first published in 1913 and 1916, dated as far back as 1902 [249]. Perhaps one of the best overviews of the state-of-the-art in the understanding of bonding in 1923 is found in the various publications arising from a meeting of the Faraday Society of the Royal Society of Chemistry in that year [250,251,252,253,254,255,256,257,258,259].

Lewis does not use the words covalent or covalency in his 1916 publication [229]. By the beginning of the 1920s, ionic and covalent compounds were being generically described as having polar or non-polar links, the latter referring to the sharing of two electrons between linked atoms [260]. Nevertheless, by 1923, Fowler [257], Lowry [255,258], and Sidgwick [260] were all using these words in more-or-less the modern sense, apparently with the assumption that the reader understood them and their context. Where did they come from? Here we have, once again, to thank Irving Langmuir [242,243,244,245,246,247], for not only did he popularize the Lewis octet model and mediate its general acceptance in the chemical community, but he also introduced a nomenclature appropriate to the model. In his first 1919 paper he writes: “It is therefore proposed to define valence as the number of pairs of electrons which a given atom shares with others. In view of the fact known that valence is very often used to express something quite different, it is recommended that the word covalence be used to denote valence defined as above” [242]. As an aside, we note that Lewis introduced the word photon in 1926 [261].

As a short summary of these sections, it is worth emphasizing that the absolutism of the early part of the 20th century C.E. in dividing bonds into ionic or covalent has now been significantly moderated and a continuum between almost purely ionic and entirely covalent (in diatomic X_2_ species) is generally recognized.

### 4.4. It’s All to do with Quantum

This article is not a history of quantum mechanics, but rather one of bonding. The fundamental development of quantum mechanics is dealt with in other excellent works [262,263,264,265,266,267,268] and we are only concerned with the transition from atoms to molecules. Nevertheless, it is not possible to underestimate the impact that the “new” quantum mechanics had on chemistry at the beginning of the 20th century C.E. It all started with Erwin Schrödinger who, after initial work on the consequences of the Bohr atom [269], published the basis of his eponymous equation in 1926.

Although exact analytical solutions could be obtained for the hydrogen atom, for larger systems with three or more particles (two or more nuclei or one nucleus and two or more electrons), it was not possible to calculate exact solutions for the Schrödinger equation. Nevertheless, the precision of the exact solutions for the hydrogen atom stimulated research into approximations for the multi-body problem.

The first published study of a polyatomic species using quantum mechanics was on dihydrogen and published by Walter Heitler and Fritz London in 1927 [270]. Heitler subsequently developed the approach to the study of homoatomic bonds and addressed the questions of ground state and valence state electron configurations [271,272,273,274]. The introduction of approximate analytic atomic wave functions for atoms other than hydrogen by researchers including John C. Slater [275], Carl Eckart [276], Linus Pauling [277], and Clarence Zener [278] was a critical advance in extending the quantum mechanical description of bonding to molecules of chemical relevance. As we see in the following section, this approach uses hydrogen-like atomic orbitals to generate localized bonds.

At the same time as the Heitler–London approach was proving scientifically and intuitively successful, an alternative quantum mechanical approach, molecular orbital theory, was being developed [279,280,281]. This approach was pioneered by Robert S. Mulliken [282,283,284,285,286,287,288,289], John Lennard-Jones [290,291,292], and Friedrich Hund [293,294] and ultimately described the bonding not in terms of localized bonds between two atoms but multi-center bonds over many atoms. The first calculations using this new method were on H_2_^+^ and H_2_.

By the end of the 1920s, two approaches were dominating—the multi-center molecular orbital approach and the localized-bonding model using hydrogen-like atomic orbitals (what we now describe as the LCAO, linear combination of atomic orbitals model). Despite the applicability of the results to chemistry, the majority of the active workers in this field came from physics. The results were regarded with great interest by the chemical community, but were probably not embraced as methods and techniques that would impinge on the practice of the working synthetic chemist. This was soon to change!

### 4.5. It’s More than just s and p—The Pauling Era and the Triumph of Valence Bonds

By the second decade of the 20th century C.E., Niels Bohr and Ernest Rutherford had developed a model for the atom comprising electrons orbiting a small central nucleus [295,296,297,298,299,300,301]. The Bohr model was predicated upon a number of assumptions: (i) electron(s) move in stable orbits around the nucleus, (ii) these stationary orbits are maintained at fixed distances from the nucleus, and (iii) the electron(s) can occupy no orbit other than these. Bohr calculated the energies of these orbits for the hydrogen atom. This allowed Bohr to correlate his calculated energies (and more critically the energy differences between his orbits) with the results obtained by spectroscopists studying the atomic spectrum of hydrogen. Rydberg [302,303], Balmer [304], and Ritz [305] had convincingly shown that the wavelengths of the lines in the atomic spectrum of hydrogen could be calculated by a simple equation involving a constant (the Rydberg constant) and two integers n_1_ and n_2_, which Bohr equated with his orbits [306]. Subsequent refinements resulted in the identification of sub-shells within each orbit with *n* > 1. Spectroscopists had introduced the descriptors sharp, principal, diffuse, and fundamental to describe lines in atomic spectra, and Hund used these, with the abbreviations *s*, *p*, *d*, *f*, to define these new subshells [307]. This is the origin of the familiar modern description of *s*, *p*, *d*, and *f* (etc.) applied to atomic orbitals [308,309].

The use of hydrogen-like atomic orbitals created a problem for the “real” chemist, who “knew” that the four C–H bonds in methane were identical. The molecular orbital method constructed different types of multicenter orbitals involving either the 2*s* or the 2*p* orbitals interacting with the hydrogen 1*s* orbitals.

**Figure 11:**
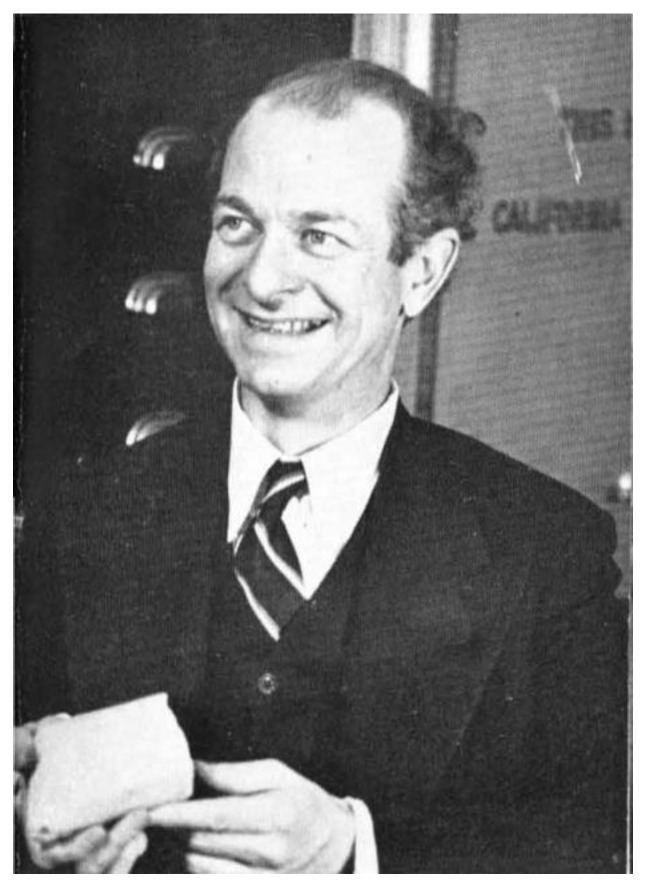
Linus Carl Pauling (1901–1994) probably did more to transform the understanding of bonding than any other scientist and the legacy of his paradigm-shift book *The Nature of the Chemical Bond* lives on today. (Public domain images taken from https://en.wikipedia.org/wiki/Linus_Pauling#/media/File:Linus_Pauling_1948.png).

The approach developed by Heitler and London evolved into valence bond theory, often described as the Heitler–London–Slater–Pauling model [310,311]. Linus Pauling (Figure 11) published his first paper on bonding in 1928 in which he correlated the Lewis model with quantum chemical calculations [312]. In 1931, he emerged as the champion of valence bond theory and explained a multitude of chemical features in theoretical terms that were also accessible and relevant to the broader chemical community. His subsequent publication *The Nature of the Chemical Bond. Application of Results Obtained from the Quantum Mechanics and from a Theory of Paramagnetic Susceptibility to the Structure of Molecules* extended the quantum mechanical treatment of the electron-pair bond to elements heavier than hydrogen [313]. Pauling also used the quantum mechanical approach to describe strong hydrogen bonds and most importantly, changing the electronic configuration of ground-state atoms to allow the generation of valence bond orbitals. For example, he postulates the change from a 2*s*^2^2*p*^2^ configuration for carbon in the ground state to 2*s*2*p*^3^ in the valence state, which is then used to construct a set of four equivalent tetrahedrally disposed eigenfunctions and uses these to explain the four equal covalent bonds in methane. He also used the valence bond approach to return the Werner model for coordination compounds. How could a transition metal bind six ligands? He started with the set of five *d* orbitals and observed that three of these (*d*_xy_, *d*_xz_, *d*_yz_) are not optimized for bond formation in octahedral complexes and subsequently constructed a set of six hybrid orbitals from the *s*, *p*_x_, *p*_y_, *p*_z_, *d*_x2–y2_ and *d*_z2_ orbitals oriented towards the vertices of an octahedron. This resulted in a consistent valence bond description of transition metal complexes. The conclusion of this paper is insightful and clearly indicates his early appreciation of the continuum between ionic and covalent bonding by stating that “the transition-group elements almost without exception form electron-pair bonds with CN^−^, ionic bonds with F^−^, and ion-dipole bonds with H_2_O; with other groups the bond type varies”. He subsequently developed idea of the unequal distribution of electrons within a chemical bond and introduced the concept of electronegativity [314]. The terms hybrid orbital and hybridization were introduced by Van Vleck in 1933 [315]. Nevertheless, the hybrid model involving metal *s, p*, and *d* orbitals was not without its difficulties. In particular, Pauling had to select between “inner” (3*d*) and “outer” (4*d*) orbitals to reproduce the electronic properties of high and low spin first row transition metal complexes.

Pauling’s vision of chemistry is best presented in the three editions of *The Nature of the Chemical Bond*, which provide not only his understanding and vision of chemical bonding, but also show how it evolved with time [316,317,318]. The hybridization model that he developed for transition metal complexes rationalized the bonding in octahedral and square-planar complexes and provided a link to crystal field theory.

The crystal field theory originated in a publication of Hans Bethe from 1929 [319] in which he investigated the influence of an electric field on an atom. He demonstrated that the terms of the atom split dependent on the symmetry of the field and the angular momentum of the atom. This was the basis of the crystal field theory subsequently developed to describe the behavior of electrons in *d*-orbitals. The model is purely electrostatic and ligands are treated as negative point charges that have a repulsive interaction with electrons in the metal atom or ion. The Bethe paper limited itself to effects in crystals and did not extend to the general case of coordination compounds. In 1932, Van Vleck extended the concept to a new crystal field theory [320] applied specifically to transition metal compounds which, on the one hand, proved to be incredibly successful but on the other hand was predicated on a physical model that completely ignored chemical bonding.

### 4.6. The Triumph of the Molecular Orbital Model in Organic Chemistry

At the same time as Pauling was developing his valence bond ideas, Erich Hückel (1896–1980) [321] was applying molecular orbital theory to unsaturated hydrocarbons [322,323,324,325]. In this work, he concentrated upon the π-bonding rather than σ-bonding in molecules and developed a model that was embraced by the organic chemistry community for its elegance, (relative) conceptual and computational simplicity, and ability to explain the properties of important unsaturated compounds such as the aromatic hydrocarbons [326,327,328]. We owe the ubiquitous and iconic picture of the doughnut-shaped electron clouds above and below the planar benzene molecule to Hückel and the success of his model. Mathematically, the Hückel approach is an LCAO method which is close to a free-electron model with the electrons moving in a one-dimensional box. Being mathematically rather simple, the basic Hückel method allowed non-computational chemists to make qualitatively accurate and chemically useful descriptions of simple unsaturated molecules. As the approach became more sophisticated, it became a powerful tool in developing theories of aromaticity and anti-aromaticity [329,330,331].

Although the great success of Hückel theory is associated with organic chemistry, the model has been extended to inorganic systems and the solid state, notably by Roald Hoffmann [332].

## 5. Quantification

### 5.1. When Calculation Moved from Pen and Paper to Machines

Early quantum mechanical calculations were performed by hand or using simple mechanical devices. The development of the electronic computer after the second world war had an enormous influence on computational chemistry. In the 1930s, Douglas Hartree was developing the use of mechanical differential analyzers, which could be applied in the numerical solution of Hartree-Fock calculations [333]. In the post-war period, one of the first electronic computers to be used for molecular quantum chemistry calculations was the Manchester Mark II, with the earliest publications appearing in the mid-1950s [334,335,336,337]. This was one of the multiple beginnings of computational chemistry in the United Kingdom. In parallel, the influence of Lennard-Jones in Cambridge ensured that the interest in computational chemistry was high. After the war, Hartree and Maurice Wilkes were ensconced in Cambridge and a thriving computational chemistry group developed, with the first publications also appearing in the mid-1950s [338,339,340,341,342,343,344,345,346]. Boys had identified the need for computational methods in a 1950 paper where he wrote with considerable perspicacity: “It is shown that the only obstacle to the evaluation of wave functions of any required degree of accuracy is the labour of computation” [347].

The United States was equally active and an overview of molecular orbital theory published by Roothaan in 1951 explained the approach and the mathematical requirements so clearly, that it was inevitable that a computational chemistry effort would develop [348,349,350]. The Roothaan paper over-shadowed a very similar manuscript published by Hall in the United Kingdom [351]. Nevertheless, the development in the United States seems to have been a little slower than in the United Kingdom [352]. Parr and Crawford in their report on the 1951 meeting *Quantum-Mechanical Methods in Valence Theory* note that, “Slater cautioned that direct attack on problems of electronic structure is not yet within the reach of automatic computing machines; the machines can do much complex arithmetic, but the problem of the molecule has not yet been reduced to an arithmetic level”, although they also give progress reports on work in Cambridge, Chicago, and other centers [353]. One of the first United States publications explicitly reporting the use of electronic computational methods is entitled the *Electronic Energy of LiH and BeH^+^* [354]. In a 1959 article entitled *Broken Bottlenecks and the Future of Quantum Mechanics*, Mulliken and Roothaan comment that a crucial step “is the programming for large electronic digital computers of the otherwise still excessively time-consuming numerical computation of these integrals, and of their combination to obtain the desired molecular wave functions and related molecular properties. The pioneering work in this field was that of Boys” and further report that “after much preliminary work in this Laboratory, we have developed a successful machine program for (a) computing accurately all the necessary integrals for diatomic molecules containing zeroth and first row atoms” [355]. This paper also shows the real impact that computers had on the field of computational chemistry as they further comment: “The importance of such a machine program is illustrated by the fact that the entire set of calculations on the N_2_ molecule which took about a year, can now be repeated in 35 min, using a machine of moderately high capacity” [355].

With the greater availability of computational resources and commercial main-frames (and subsequently personal computers, clusters and super-computers), the area of computational chemistry blossomed to the present state, where complex calculations at the DFT or other levels (see the next section) can be carried out on a laptop using commercial programs.

### 5.2. Semi-Empirical Methods

One of the greatest influences of the availability of low-cost computational capacity was the acceptance by the chemical community that non-exact quantum chemical solutions could be of great benefit to the wider community. This resulted in the development of a whole range of semi-empirical methods, of which the Hückel model could be seen as the progenitor. Hoffmann further developed the Hückel approach into his extended Hückel method in which he considers not only the π-bonding but also the σ-bonding in the system [356,357].

As computational capacity became available, it was possible to return to methods that had been considered but rejected as calculationally too demanding in the past. The majority of the high-level calculations on multiatomic systems in the 1960s and 1970s are based on the Hartree–Fock self-consistent field method. This was first formulated by Hartree in 1927 (published 1928) as a method for calculating approximate wave functions and energies using *ab initio* methods [358]. The Hartree approach was criticized by Slater [359] and Fock [360,361] in terms of the antisymmetry of the wave function. Summarizing the Hartree–Fock approach, the Born–Oppenheimer approximation is obeyed and the nuclei are treated as static, relativistic effects are completely neglected and each energy eigenfunction is described by a single Slater determinant.

The semi-empirical methods attempt to address some of the deficiencies of the computationally demanding Hartree–Fock approximation for the determination of the stationary state wave function and energy of multi-body systems. They are driven by the competing desires on the one hand to simplify the computational demands, and on the other hand to increasing the speed and improving the accuracy of the calculations.

It is now time to enter into the world of acronyms by which many of these methods are known. These methods had their heyday in the last quarter of the 20th century C.E. but are still used when either a high level of accuracy or, paradoxically, computationally cheap and easy calculations are required. Most methods are based upon the neglect of diatomic differential overlap (NDDO), which allows simplification of the Hartree–Fock equations. A widely used implementation of the NDDO method was in the modified neglect of diatomic overlap (MNDO) approach introduced by Dewar in 1977 [362], but numerous related approaches including complete neglect of differential overlap (CNDO, 1965) [363,364,365] intermediate neglect of differential overlap (INDO, 1967) [366], modified intermediate neglect of differential overlap (MINDO, 1975) [367], and Zerner’s intermediate neglect of differential overlap (ZINDO, 1973) [368] were also developed.

Slightly different approaches were implemented in the Austin Model 1 (AM1, 1985) [369] model, which resembled MNDO but treated the nuclear-nuclear repulsion in a different manner and the parametric method 3 (PM3, 1989) [370,371,372,373], which is essentially a reparameterization of AM1.

### 5.3. Density Functional Theory

Today (2020), density functional theory (DFT) is one of the most common methods used for studying chemical systems [374,375,376,377]. The *ab ibitio* methods described above are still widely used and have advantages for some systems. However, and possibly more than any other advance in computational chemistry, DFT has made the synthetic chemist realize that quantum chemical methods are a tool as valuable in planning chemical transformations as spectroscopy and crystallography are in determining the nature of their products. The insight that DFT gives into chemical bonding is profound and has had a genuinely paradigm-shifting impact on chemistry. DFT is based upon the use of exchange-correlation functionals that use electron density to describe the many-body effects within a single particle formalism. At the core of the DFT method is the Hohenberg–Kohn theorem. The contribution of Walter Kohn was honored by the award of the 1998 Nobel Prize in Chemistry for the ‘‘development of the density-functional theory’’. The precise description of DFT as a calculational method is still debated, as is the degree and accuracy of the calculation of electron correlation [378,379,380,381,382].

For *n*-electron systems, exact quantum mechanical solutions can only be classically obtained for hydrogen atoms and helium monocations [383]. Hohenberg and Kohn proposed that the exact ground-state energy can be described as a function of a function (a functional) relating to the ground-state one-particle density [384,385]. All that remained was to develop appropriate functionals! Once these were available, the goal describing motions and pair correlations of a many-electron system by the total electron density became a seductive challenge. Kohn and Sham showed that ground state self-consistent equations could be developed that were analogous to the Hartree and Hartree–Fock descriptions. The exchange and correlation parts of the chemical potential of an electron gas are treated as additional effective potentials.

The model originated in physics and it was originally thought that it could not be applied to the detailed analysis of chemical systems at the atomistic level. However, relatively early in the development, it was shown that appropriate functionals gave good descriptions of the geometry and dissociation energy of molecules. Subsequently, the development of DFT allowed accurate prediction of energies along a reaction coordinate and the identification of reaction intermediates and transition states. Today, not only is DFT used to investigate the ground state properties of molecular systems, but also to study their excited state properties and the transition states and intermediates along a reaction coordinate. The increasing use of time-dependent DFT methods is allowing a profound insight into the behavior and properties of excited state species [386,387,388,389].

### 5.4. And Back to the Simplicity of Bonds—The Natural Bond Order Approach

Despite the success of molecular orbital theory, chemists were often left seeking the simplicity of the Lewis and valence bond descriptions of two-electron two-center bonds rather than the complexity of multi-center delocalized descriptions of bonding. In 1955, Löwdin introduced the concept of “natural orbitals” [390]. Natural bond orbital methods seek to represent electronic wavefunctions in terms of localized Lewis-like chemical bonds [391,392]. The computational method has been incorporated in many of the standard quantum chemical packages used in chemistry and analysis in terms of natural atomic orbitals, natural hybrid orbitals, natural bonding orbitals, and natural (semi-)localized molecular orbitals is common in some areas of chemistry. The approach has been adopted with various degrees of enthusiasm, although it appears very versatile and intuitive and has been extended to periodic systems, bulk solids, and surfaces [393]. Extension of the concept to hydrogen-bonding has proved to be more controversial [394,395,396].

One interesting development of the NBO approach is found in Boldyev’s adaptive natural density partitioning (AdNDP), which combines the simplicity of Lewis theory with the broader appeal of molecular orbital theory [397]. Chemical bonds are treated as *n*-centre 2-electron bonds, where *n* can vary from one (a lone-pair), through two (a conventional Lewis two-electron bond) to the maximum number of atoms in the system (completely delocalized bonding). This approach has found considerable success in describing the bonding in inorganic and main-group cluster compounds [398,399].

## 6. The Challenge Bonding

By the beginning of the 1960s, all was well in the chemical universe. Chemists understood and ordered materials according to the extreme descriptions of covalent two-electron two-center bonds and ionic charge-separated. Of course, delocalized bonding was understood and invoked when necessary and coordination compounds produced a conundrum falling somewhere between the two extremes. In the next half century, many of these conceptions and pre-conceptions were to be challenged, modified, discarded or replaced in the light of ever more interesting and challenging compounds being prepared and characterized.

### 6.1. Metals Bowl a Googly

In the language of the cricketer, just when you think you understand something, Nature has the tendency to bowl you a googly. The organic community had long accepted C–C multiple bonds, but inorganic chemists were blind-sided in the 1960s by the discovery of metal–metal multiple bonds [400]. How much of the initial skepticism was based in the memories of the Jørgensen chain theory is conjecture. Nevertheless, the metal–metal quadruple bond served to remind the community that metals still had a few tricks up their sleeves [401]. Some forty years later, the inorganic community was again surprised by compounds containing metal–metal quintuple bonds [402,403,404,405,406]. Even sextuple bonds have been identified in dimolybdenum (Mo_2_) and ditungsten (W_2_) [407,408,409,410,411,412]. These new types of bonds stimulated an enthusiastic and sometimes heated debate about the correlations between bond strength, bond order, and bond length. The novel *The Delta Star* by Joseph Wambaugh, is possibly the only work of fiction to feature Harry Gray and the spectroscopy of metal–metal multiply-bonded compounds [413].

### 6.2. When is a Bond not a Bond?

The modern chemist is confronted with an ever more complex and interesting array of types of bonding. There is an emerging and increasing interest in interactions that lie outside the classical Lewis two-electron two-center model, but which nevertheless find atoms closer together than their van der Waals radii would suggest.

The commonest example of this type would be the classical hydrogen bond, which is well-rationalized in models and calculational approaches involving multi-center bonding [414]. The concept of the classical hydrogen bonds dates back to Alfred Werner, who depicted ammonium chloride as NH_3_…HCl [415] and was revived by Latimer and Rodebush in their use of Lewis model to explain the anomalous properties of water [248]. The hydrogen bond was an integral part of the coherent views of molecular structure promulgated by Linus Pauling [316,317,318].

The increasing routine availability of crystallographic methods to characterize solid state materials, and in particular, the accessibility of databases and the use of artificial intelligence to analyze large sets of structural data, have allowed the identification and establishment of non-classical hydrogen bonds such as C–H…O, although these were first postulated in 1937 [416,417]. Once again, interactions of this nature have stimulated the debate about distance and energetics, although in most cases the “bond” energies are relatively low (4–5 kJ mol^−1^) [418,419,420].

Metals can also develop attachments to neighbors, and the term metallophilic was introduced in 1994 by Pekka Pykkö [421] as a general description of a phenomenon in which compounds with multiple metal centers exhibiting *d*^8^, *d*^10^, and *s*^2^ outer shell configurations often show short (close to the sum of the atomic radii) contacts between metal centers, indicative of an attractive interaction rather than the expected repulsive ones. The description metallophilic was a general extension of the phenomenon of short Au(I)…Au(I) interactions in a large number of compounds and termed aurophilicity by Pykkö and others [422,423,424,425,426,427]. In gold compounds, the aurophilic interaction is typified by Au…Au distances close to 3 Å. Pykkö described the aurophilic interaction as amongst the strongest closed-shell interactions, with bond energies of 10–50 kJ mol^−1^ approaching those of weak covalent bonds or hydrogen bonds, although the word aurophilic was only coined in 1988 by Schmidbaur.

The general approach of investigating short interactions between molecules in the solid state has recently been described as the quantum theory of atoms in molecules (QTAIM) model [428,429,430,431]. QTAIM was originally seen as providing a physical justification for the Lewis bonding model and the pairing of electrons together with electron localization lie at the core of the description, but is evolving beyond that first vision. Bader, the originator of the QTAIM model, has also made the correlation with molecular orbital theory [432]. Closely related to QTAIM is the electron localization function (ELF), which is the probability of finding an electron in the neighborhood of a reference electron at a given point and possessing the same spin. The ELF not only shows a separation between the core and valence electrons according with chemical intuition, but also a visualization of covalent bonds and lone pairs [433].

It seems appropriate to end our scientific journey here, with methods that are beginning to provide a direct visualization of bonds.

## 7. Conclusions and Reflections

We have made a long journey from ancient Greece to modern bonding theory. It is certainly not a journey that has reached its end. We will finish our part of the journey with the words of Roald Hoffmann (as quoted by Philip Ball in an article entitled *Beyond the Bond* [434]): “Any rigorous definition of a chemical bond is bound to be impoverishing” and his advice to “have fun with the fuzzy richness of the idea”. Enjoy the fuzziness of the bond, think occasionally of the little hooks and do not let dogma or conventional views of bonding limit your creativity.
